# Machine-Learning Classification of Solids Mass Flow Rate in Gas–Solid Pneumatic Conveying Using Electrostatic Sensor Measurements

**DOI:** 10.3390/s26217085

**Published:** 2026-11-09

**Authors:** Özcan Kamışlı, Mehmet Bilginer Gülmezoğlu

**Affiliations:** Department of Electrical and Electronics Engineering, Eskişehir Osmangazi University, 26040 Eskişehir, Türkiye

**Keywords:** electrostatic sensing, electrostatic sensor array, mass flow rate classification, data-driven measurement, common vector approach, convolutional neural network, support vector machine, cross-condition validation, feeding-cycle hold-out

## Abstract

Electrostatic signals in pneumatic conveying depend on solids mass flow rate, air velocity, particle distribution and material, complicating direct estimation. This study classified five nominal solids mass flow rates (0.1–10 g/s) for wheat flour and ground coffee at four air velocities (15–30 m/s) using six ring electrodes and seven arc segments with guarded electrometer-grade front ends. From 1 s blocks, 29 time-, frequency- and time–frequency-domain features per channel were extracted. The Common Vector Approach (CVA), support vector machines (SVMs), multilayer perceptrons (MLPs), random forests (RFs) and feature-based convolutional neural networks (CNNs) were compared with an end-to-end CNN on raw signals. With one feeding cycle per record held out, the best balanced accuracies were 86.9% (flour) and 89.6% (coffee), and 97.8–100% of predictions were within one class. RF regression yielded typical error factors of 1.34 (flour) and 1.31 (coffee). Depending on the classifier, balanced accuracy declined to 47–70% for unseen air velocities and 26–59% across materials; a no-flow control suggested possible contributions from record-specific offsets to the accuracy. These findings restrict the reported performance to the tested conditions and support velocity-range coverage, material-specific calibration and recording-order controls.

## 1. Introduction

Pneumatic conveying transports bulk solids through enclosed pipelines in cement, power, steel and food industries, allowing automation and low-maintenance operation [1]. Online solids mass flow measurement supports process control and the reduction in particle emissions and waste. Non-uniform solids distribution and velocity, pipe geometry and deposits, and material properties such as size, shape, composition and moisture content complicate the measurement [2]. Acoustic, Coriolis, capacitive, thermal, optical, differential-pressure, electromagnetic and electrostatic techniques have been applied to this measurement [2,3,4].

Electrostatic sensors offer passive operation, low cost, high sensitivity, a simple and robust structure, easy installation, and low-maintenance monitoring even with simple electrodes [5]. Particles generate charge through collisions and friction, typically $10^{-7}$–$10^{-3}$ C/kg; this charge induces a signal on an insulated electrode (Section 2.1) [6]. The induced response depends on charging conditions and electrode geometry, so it cannot be predicted precisely [7,8]. A single ring electrode integrates signals over the cross-section and, therefore, poorly resolves solids distribution.

An electrostatic sensor consists of the electrodes and a signal conditioning circuit (SCC). The electrodes are usually metallic rings or arc segments mounted flush with, and insulated from, the grounded pipe wall [9], although rod and plate electrodes and intrusive designs are also used [2,10]. Here, the electrodes are referred to as flow noise antennas (FNAs), a term coined in the preliminary analysis of these data [11]. The induced currents range from femtoamperes to nanoamperes and are highly susceptible to electromagnetic interference, so the SCC, above all its analogue front end (AFE), is a critical part of the measurement chain [12].

Ring, arc and rod electrode arrays have extended electrostatic sensing beyond cross-correlation-based particle velocity measurement to concentration and flow-pattern characterisation [2,10,13]. An eight-arc array and an artificial neural network (ANN) identified four pulverised-coal flow patterns [14]; later methods used cross-rod arrays and deep residual networks [15,16]. Flow-pattern classification should be distinguished from the measurement of solids mass flow rate with ring–arc arrays [11,17].

Because the induced signal depends on particle charge and velocity as well as on the solids mass flow rate [7], the rate is increasingly estimated with data-driven methods that learn this relation from measured signals. Approaches progressed from signal statistics and power spectral density with principal component analysis and neural networks [18,19] to support vector machines (SVMs), convolutional neural networks (CNNs), spectral mappings and raw-signal networks, including a study with held-out operating conditions [17,20,21,22,23]. The closest ring–arc study and most of this literature address continuous rates above about 2 g/s, usually for one material (Section 4). Transfer across pipe conditions or materials has rarely been evaluated and has sometimes yielded poor results [4,24].

Discrete solids mass flow classes support operating-band monitoring in dosing, blockage prevention and fault detection, particularly when low-flow signals approach the no-flow level, where a reliable coarse estimate may be more attainable than an accurate regression. Because particle velocity, concentration, distribution and charging jointly affect the response (Section 2.1), different flow-rate–velocity combinations produced similar signals here (Section 3.3). Flow-pattern variation may add uncertainty [14], but the nominal dilute-phase regime was not independently characterised (Section 3.1 and Section 4). Unlike approaches combining electrostatic, differential-pressure, capacitive or acoustic sensing [17,21,24,25,26], a ring–arc array offers complementary spatial responses for discrete classification within a single electrostatic sensing modality (Section 3.5).

This study classifies the solids mass flow rate into five nominal levels (0.1–10 g/s) from a ring–arc electrostatic array, with feeding cycles, air velocities and materials held out during validation; to the best of the authors’ knowledge, such a classification at flow rates down to 0.1 g/s has not been reported. Its contributions are (i) six ring and seven arc channels with guarded electrometer-grade front ends and a measured input-referred no-flow root-mean-square (RMS) level of approximately 18–45 pA; (ii) 798 one-second blocks for wheat flour and ground coffee at four air velocities, with batch-weighed reference rates; (iii) a comparison of the Common Vector Approach (CVA), first applied to electrostatic flow measurement in the preliminary analysis [11], with SVMs, multilayer perceptrons (MLPs), random forests (RFs), feature-based CNNs and an end-to-end CNN on raw signals under random 8-fold cross-validation, feeding-cycle hold-out and leave-one-velocity-out and (iv) an assessment of electrode types, cross-material transfer, record-specific no-flow offsets, classification versus regression and computational cost. These tests define the conditions under which the method is valid.

## 2. Materials and Methods

### 2.1. Measurement System and Sensing Principle

**Figure 1:**
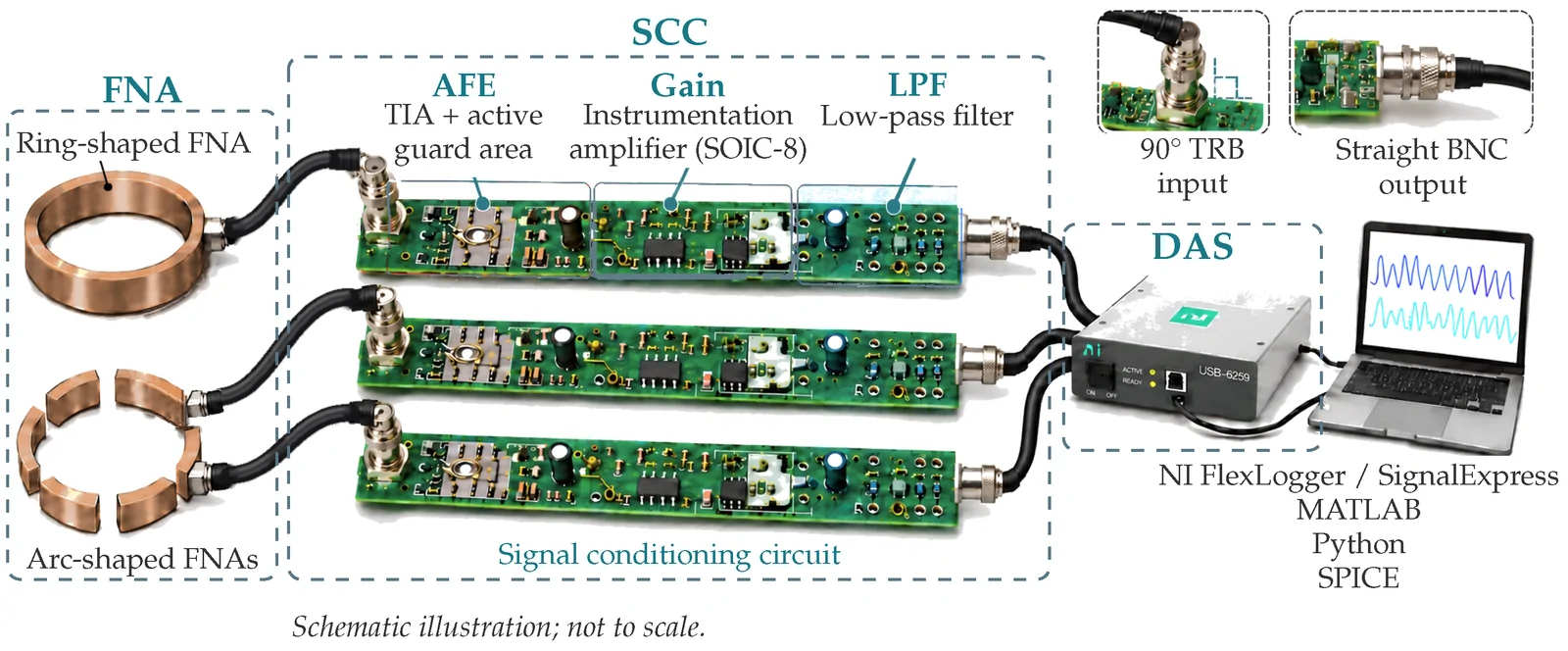
Overall measurement system from the flow noise antenna (FNA) electrodes to the computer; AFE, analogue front end; DAS, data acquisition system; insets: triaxial bayonet (TRB) input and Bayonet Neill–Concelman (BNC) output connectors.

Figure 1 depicts the measurement system; as representative examples, one ring and two arc electrodes are shown. Each FNA used for classification is connected through an actively guarded triaxial cable to the triaxial bayonet (TRB) input of its own SCC, which comprises the AFE, a gain and level-shifting stage and a low-pass filter. The conditioned output reaches a Bayonet Neill–Concelman (BNC) connector, is digitised with a multichannel data acquisition system (DAS) and is recorded on a computer.

**Figure 2:**
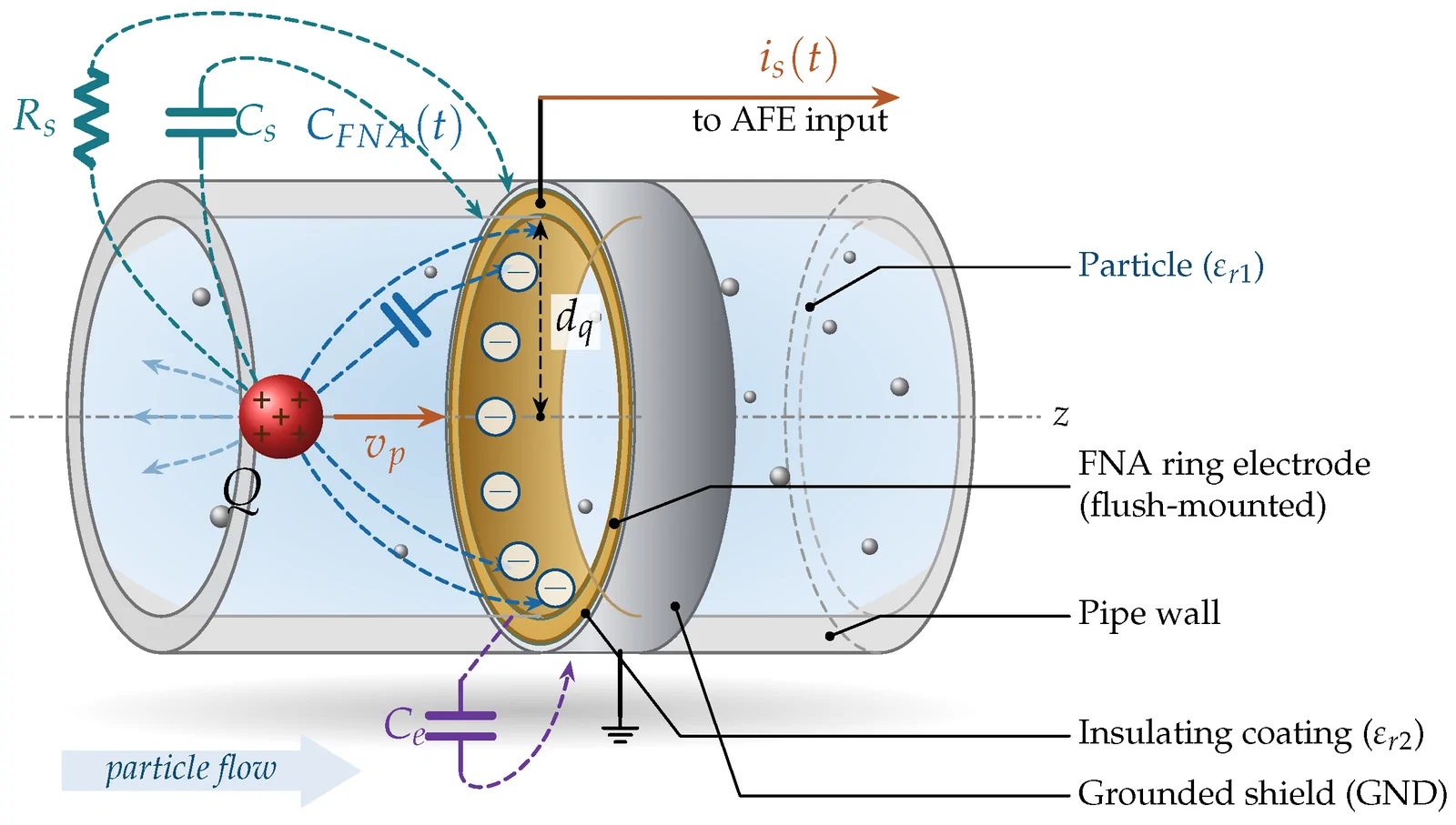
Sensing principle of a flush-mounted FNA ring electrode and its equivalent circuit at the analogue front end (AFE) input: particle charge *Q*, velocity $v_{p}$ and radial distance $d_{q}$; induced current $i_{s}\left(t\right)$; time-varying coupling capacitance $C_{FNA}\left(t\right)$; insulation resistance $R_{s}$; shunt capacitance $C_{s}$; electrode-to-shield capacitance $C_{e}$ (included in the input capacitance $C_{i}$ of the circuit model in Section 3.2); relative permittivities $\varepsilon_{r1}$ (particle) and $\varepsilon_{r2}$ (insulating coating).

For a given electrode geometry, the RMS value of the conditioned signal depends on the process and material quantities as

(1) $$V_{\mathrm{rms}}≅f\left(Q_{m},R_{as},v_{p},\mathrm{RH},d_{p},\rho_{p},\sigma_{p},\varepsilon_{p}\right),$$

where $Q_{m}$, $R_{as}$, $v_{p}$, RH, $d_{p}$, $\rho_{p}$, $\sigma_{p}$ and $\varepsilon_{p}$ are the solids mass flow rate, air–solids ratio, particle velocity, relative humidity, particle diameter, density, conductivity and permittivity, respectively [27,28]. For one material under stable ambient conditions, the last five quantities are approximately constant and $V_{\mathrm{rms}}$ is governed by $Q_{m}$, $R_{as}$ and $v_{p}$, which are coupled through the air flow and act jointly on particle charge, concentration and pulse shape, so one RMS value cannot separate a change in $Q_{m}$ from a change in $v_{p}$. The underlying field obeys Poisson’s equation with boundary conditions on the grounded pipe $\Gamma_{p}$, the insulating coating $\Gamma_{s}$ and the electrode $\Gamma_{e}$ (Equation (2)), where $\nabla^{2}$ is the Laplace operator, ϕ(r,θ,z) the potential in the pipe, $\rho_{q}$ the charge density of the particles, $\varepsilon_{0}$ the permittivity of free space and *c* the uniform potential of the electrode surface. The charge induced on the electrode is the flux of this field through $\Gamma_{e}$ (Gauss’s law), and its time derivative is the current $i_{s}\left(t\right)$ (Figure 2). Because $\rho_{q}$ varies with the number, charge and trajectories of the particles, $i_{s}\left(t\right)$ is non-stationary and carries information on the solids concentration, cross-sectional distribution and velocity.

(2) $$\left\{\begin{array}{c} \nabla^{2}\phi =-\frac{\rho_{q}}{\varepsilon_{0}}\\ {\phi \left(r,\theta ,z\right)|}_{\left(r,\theta ,z\right)\in \Gamma_{p}}=0\\ {\phi \left(r,\theta ,z\right)|}_{\left(r,\theta ,z\right)\in \Gamma_{s}}=0\\ {\phi \left(r,\theta ,z\right)|}_{\left(r,\theta ,z\right)\in \Gamma_{e}}=c \end{array}\right.$$

For a flush-mounted FNA (Figure 2), a particle with charge *Q* moving with velocity $v_{p}$ through its sensing volume induces a transient image charge of opposite sign, returning to zero as the particle moves away; induction rather than direct charge transfer dominates. The electrode acts as a current source $i_{s}\left(t\right)$ at the AFE input, coupled to the particle through the time-varying capacitance $C_{FNA}\left(t\right)$, which depends on the axial position of the particle and on its radial distance $d_{q}$ from the electrode, so that the amplitude and duration of each current pulse depend on *Q*, $d_{q}$ and $v_{p}$. The source is loaded by the insulation resistance $R_{s}$, the shunt capacitance $C_{s}$ and the electrode-to-shield capacitance $C_{e}$ across the insulating coating of relative permittivity $\varepsilon_{r2}$; $\varepsilon_{r1}$ is that of the moving charged dielectric particle.

### 2.2. Feature Extraction

The signal reflects several coupled mechanisms, such as particle–wall collisions, roping and variations in solids concentration and velocity, so changes in the flow may appear as amplitude or distribution shifts in the time domain or as a redistribution of energy between frequency bands. Therefore, 29 time-, frequency- and time–frequency-domain features were extracted from each 1 s block of each channel (Table 1). Apart from the RMS value and the cross-correlation used for velocity measurement, only some of these descriptors have been related to the flow parameters [17,18]; here, all 29 serve as inputs to the data-driven models.

For time-domain features 1–16, the RMS, standard deviation, variance and simple square integral quantify the magnitude and energy of the fluctuations. After mean removal and clipping at ±3 standard deviations, they are closely related, and the mean reflects only the asymmetry introduced by the clipping. Skewness and kurtosis characterise amplitude asymmetry and tails, reflecting non-Gaussian, impulsive behaviour; the peak-to-peak value and the peak, impulse and clearance factors describe the dynamic range relative to the RMS or mean absolute level. Zero crossings, slope-sign changes and waveform length reflect oscillatory activity; the Willison amplitude counts sample-to-sample changes above a threshold, and the entropy of the amplitude histogram measures irregularity. Frequency-domain features 17–21, the peak amplitude, total power, mean frequency, spectral entropy and shape factor, describe the dominant component, energy, average frequency, bandwidth character and spread of the spectrum. Time–frequency features 22–29 are computed from the short-time Fourier transform (STFT) of the block (Hann window of 256 samples, 50% overlap); the RMS, mean and variance of the STFT magnitude quantify the time–frequency energy and its variability. The mean-crossing irregularity, the coefficient of variation of the STFT magnitude, measures how unevenly this energy is distributed, and four quantile-frequency ratios describe the spectral bandwidth.

### 2.3. Data-Driven Models and Validation

**Figure 3:**
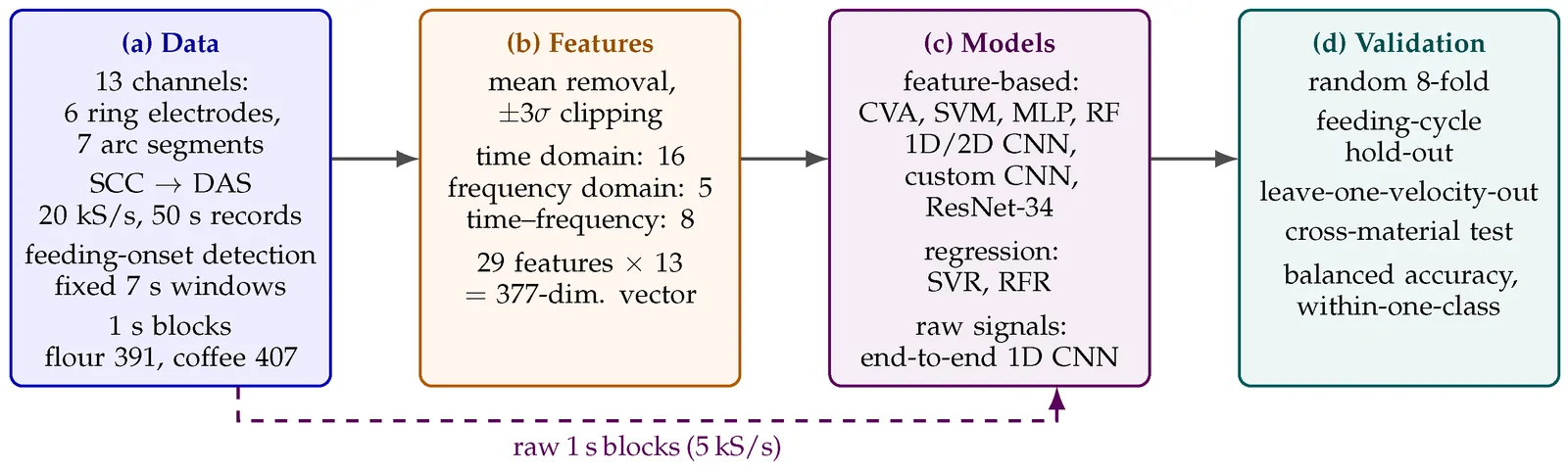
Measurement and data-driven workflow: (**a**) data acquisition and block construction; (**b**) feature extraction; (**c**) feature-based and end-to-end classifiers and regressors; (**d**) validation protocols and metrics; dashed arrow: raw-signal branch bypassing the feature extraction.

Four classifier families with different modelling assumptions were compared using identical feature inputs: CVA, a subspace method suited to few training vectors per class; SVMs with linear and radial basis function (RBF) kernels; an RF and neural networks, namely an MLP and one- and two-dimensional (1D and 2D) CNNs. For comparison with learned representations, the custom CNN of the preliminary analysis [11], an ImageNet-pretrained ResNet-34 and an end-to-end CNN on the raw signals were also evaluated, and for comparison with continuous estimation, support vector and random forest regressors (SVR, RFR) were trained on $\log_{10}Q_{m}$. Models were implemented in Python 3.14: CVA with NumPy 2.4.6; SVM, SVR, MLP, RF and RFR with scikit-learn 1.9.0 and the CNNs with PyTorch 2.14.1 (Figure 3).

CVA represents each class *c*, with training vectors $a_{1}^{c},\ldots ,a_{m_{c}}^{c}$, by a common vector $a_{\mathrm{com}}^{c}=a_{i}^{c}-P_{c}\;a_{i}^{c}$, obtained by removing from a training vector its projection onto the class difference subspace $B_{c}=\mathrm{span}{\left\{a_{k}^{c}-a_{1}^{c}\right\}}_{k=2}^{m_{c}}$; $P_{c}$ is the orthogonal projection onto $B_{c}$, and $a_{\mathrm{com}}^{c}$ is the same for every training vector of the class [29,30]. In the insufficient-data case, where the feature dimension (377) exceeds $m_{c}$, a test vector x is projected onto the orthogonal complement of each class difference subspace and assigned to the class whose common vector is nearest, $\hat{c}=\arg\min_{c}∥x-P_{c}\;x-a_{\mathrm{com}}^{c}∥$; by construction, every training vector is then classified correctly.

Each binary SVM assigns a block x to one of two classes by the sign of $f\left(x\right)=\sum_{i}\alpha_{i}y_{i}K\left(x_{i},x\right)+b$, where the support vectors $x_{i}$ are training blocks with labels $y_{i}=\pm 1$ and non-zero coefficients $\alpha_{i}$, *K* is the kernel and *b* the offset; the coefficients are found by maximising the margin, with the regularisation parameter *C* penalising margin violations, and the five classes are separated by one-versus-one voting over ten binary SVMs. The linear SVM used $K\left(x_{i},x\right)=x_{i}^{T}x$ with C=1, and the RBF-SVM used $K\left(x_{i},x\right)=\exp\left(-\gamma {∥x_{i}-x∥}^{2}\right)$ with C=10 and γ=1/377, the inverse of the feature dimension; both used class-balanced weights to compensate for the smaller number of 0.1 g/s blocks. The MLP had two hidden layers of 256 and 64 rectified linear units (ReLU) with an L2 penalty of $10^{-3}$; the RF consisted of 500 class-balanced trees; the SVR used the same RBF kernel and the RFR used 500 trees. For all feature-based models, including CVA, features were standardised using training-only statistics, and all hyperparameters were fixed in advance and not tuned on the test data.

**Figure 4:**
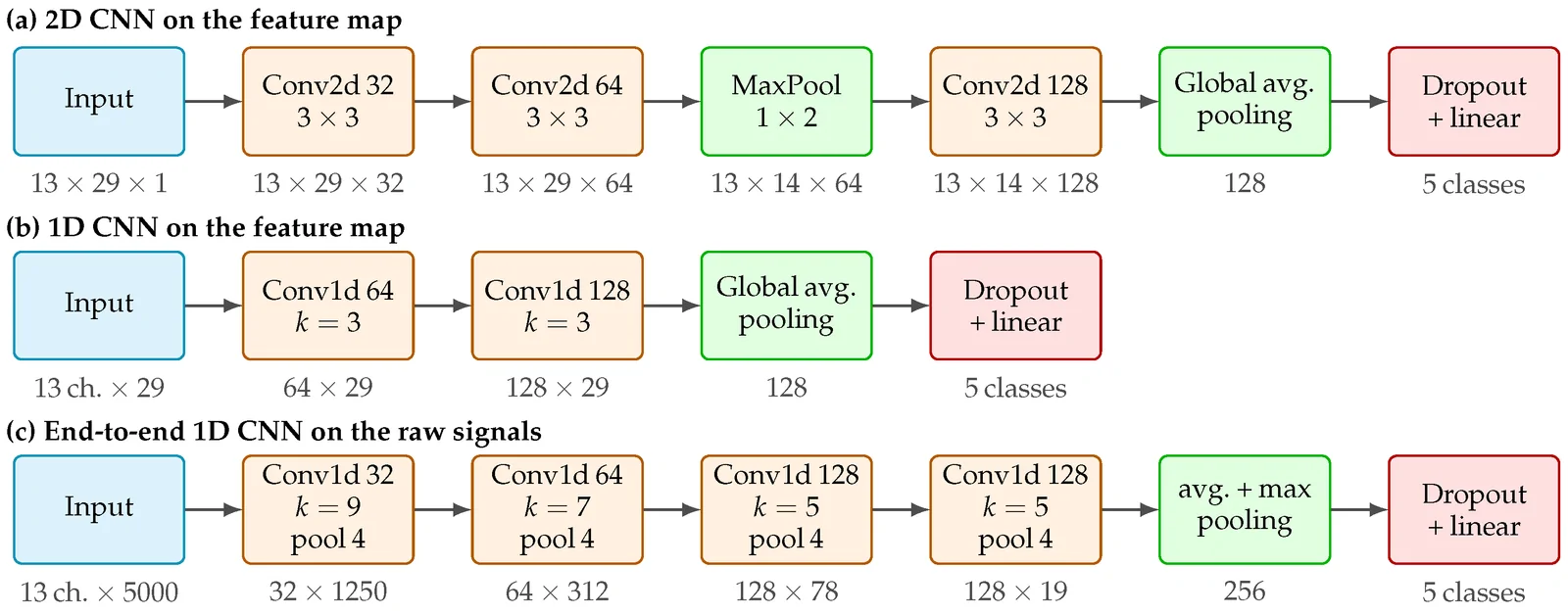
Convolutional neural network (CNN) architectures and their inputs: (**a**) 13×29 electrode-by-feature map; (**b**) the 13 electrodes as input channels along the feature axis; (**c**) raw 1 s blocks decimated to 5 kS/s. Each convolution is followed by batch normalisation and a rectified linear unit (ReLU).

CNNs learn local patterns through shared convolution kernels [31]. For the 1D and 2D CNNs, each 377-dimensional vector was arranged as a 13×29 map with electrodes as rows and features as columns, so that convolutions combine neighbouring features and electrodes of the same block (Figure 4a,b); both were trained for 150 epochs with class-weighted cross-entropy and the AdamW optimiser (constant learning rate $10^{-3}$, weight decay $10^{-3}$, batch size 32, dropout 0.3). For the custom CNN and the ResNet-34, the map was bilinearly resized to 224×224 and, for the ResNet-34, copied to the three input channels; with a batch size of 16, the custom CNN was trained for 100 epochs at a learning rate of $10^{-3}$ and the ResNet-34, with all layers fine-tuned, for 30 epochs at $10^{-4}$. The end-to-end 1D CNN (Figure 4c) was trained on the raw 1 s blocks of the 13 channels, decimated to 5 kS/s after anti-alias filtering, for 60 epochs with a cosine learning-rate schedule and random 4096-sample crops; the global pooling of the last layer allows the full 5000-sample blocks to be used at test time.

The 1 s blocks of each material (Section 3.3) were split under three validation protocols: (i) random stratified 8-fold cross-validation over blocks, in which blocks of the same feeding cycle appear in both training and test sets; (ii) feeding-cycle hold-out, in which two feeding cycles of every test condition are used for training and the third for testing, rotated over the three cycles, so that training and test data never share a signal segment, although they come from the same 50 s record (Section 3.6) and (iii) leave-one-velocity-out, in which all blocks of one air velocity are held out to assess generalisation to an unseen operating condition. Models trained on one material were also tested on the other (cross-material test). All models shared the same splits; MLP, RF and the 1D and 2D CNNs were trained with five random seeds and the custom CNN, ResNet-34 and end-to-end CNN with three. Because the classes are imbalanced, the balanced accuracy is reported together with class-wise precision, recall and F1 scores and the proportion of predictions within one class of the true class.

## 3. Experimental Studies and Results

### 3.1. Test Rig and Test Conditions

**Figure 5:**
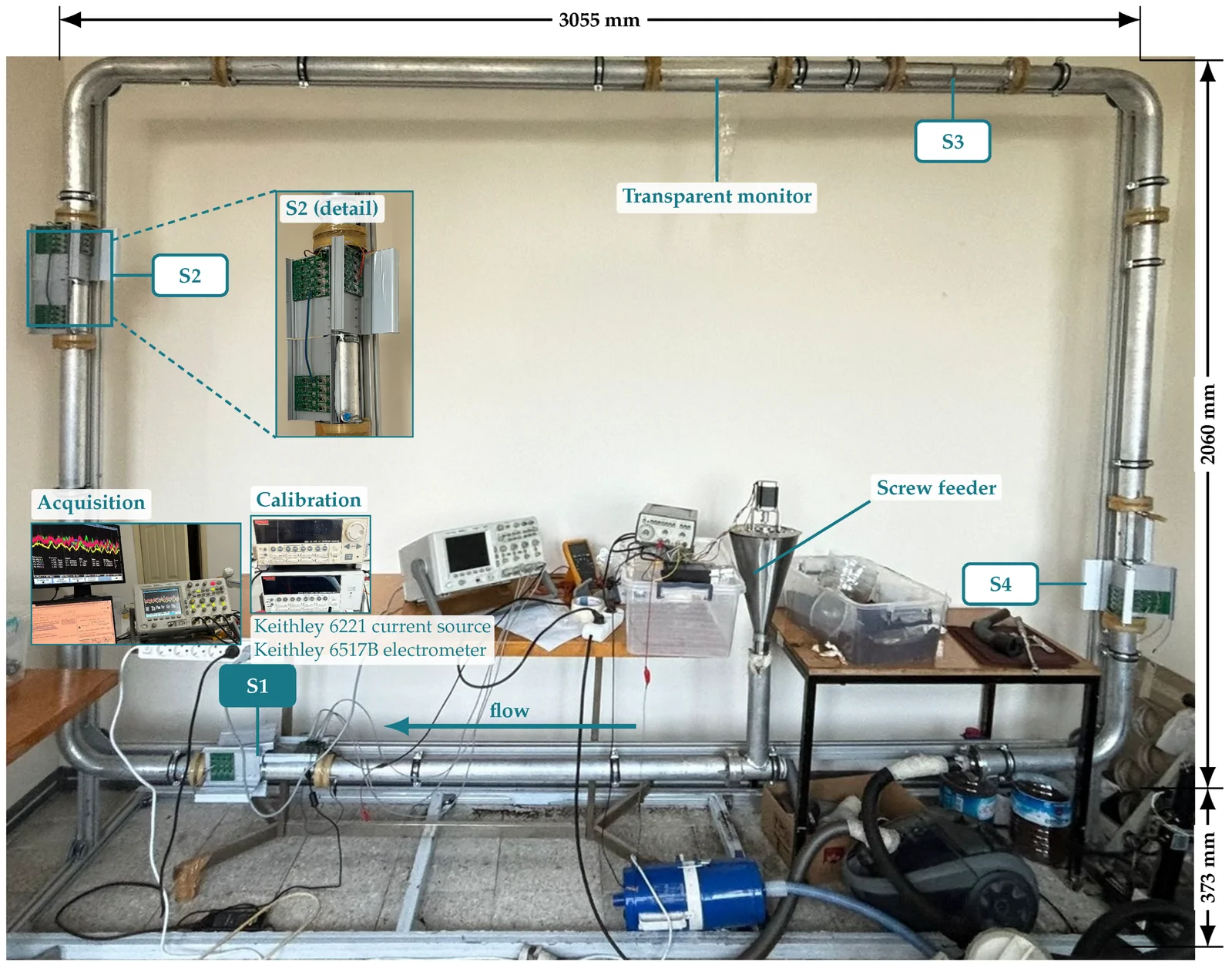
Laboratory-scale pneumatic conveying test rig with electrostatic sensors S1–S4 (perspective-corrected photograph); dimension lines: nominal outer dimensions of the pipe loop and height of the supporting frame; dashed lines: magnified view of sensor S2; arrow: flow direction downstream of the screw feeder.

The laboratory-scale test rig (Figure 5) comprises a vacuum pump and a screw feeder, each driven by a variable-frequency drive (VFD), and a closed-loop metal pipeline of inner diameter D=50 mm carrying four electrostatic sensors (S1–S4). All measurements used S1, in the lower horizontal section about 1.5 m (30D) downstream of the feeder inlet and 0.5 m (10D) upstream of the next bend. Wheat flour (bulk density 0.6 g/cm^3^, average particle diameter 200 µm) and ground coffee (0.4 g/cm^3^, 300 µm) were conveyed in separate series; both are Geldart group B powders [32,33] but differ in composition, density and particle size, and hence in triboelectric charging. At air velocities of 15–30 m/s, the air mass flow rate is about 34–68 g/s and the solids-to-air mass loading ratio, the inverse of $R_{as}$ in Equation (1), is about 0.001–0.3. These values are consistent with nominal dilute-phase operation, although the regime was not independently characterised. The laboratory was air-conditioned (average 30 °C, 36% relative humidity).

Each test condition (TC) combines a nominal mass flow rate $Q_{m}$ with a constant air velocity $V_{s}$, set with the VFD of the vacuum pump and measured with an anemometer (uncertainty of ±1%). Five rates (0.1, 0.5, 1, 5 and 10 g/s; classes $C_{1}$–$C_{5}$) and four velocities (15, 20, 25 and 30 m/s) give 20 TCs per material (Table 2); one flour record (0.1 g/s at 20 m/s) showed no measurable flow and was excluded. The rates were selected from a wider programme of 23 (flour) and 25 (coffee) rates between 0.01 and 50 g/s (not all at every velocity) to span two decades at the low end of the dilute-phase range, where electrostatic signals approach the noise floor of conventional front ends. Velocities below 15 m/s were not used: below about 10 m/s the powders settled in the pipe, consistent with an earlier study [17] in which higher solids mass flow rates were unworkable at low air velocities. For every TC, three batches of the mass delivered in 10 s at $Q_{m}$ (e.g., 10 g for 1 g/s) were weighed (1 mg resolution) and discharged in turn by the screw feeder, each within 10 s, giving a 50 s record of three feeding cycles separated by no-flow intervals. Because vibration and local powder accumulation make the instantaneous discharge non-uniform, $Q_{m}$ is a 10 s average; the 10 s period allows the flow regime to become established within each cycle. Repeatability had been checked with repeated records in preliminary series on the same rig; the dataset analysed here uses one record per TC.

### 3.2. Sensor and Signal Conditioning

**Figure 6:**
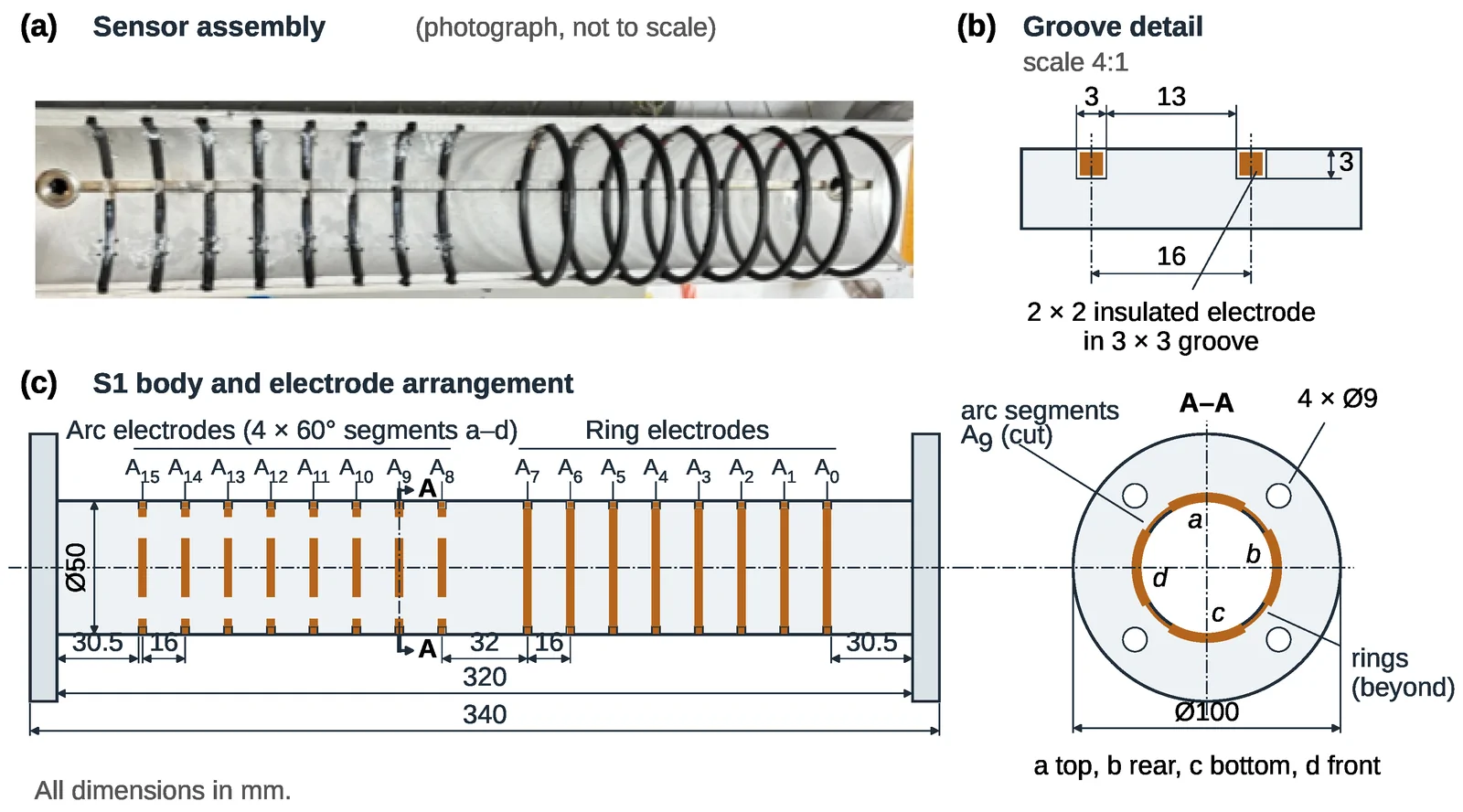
Electrostatic sensing unit S1: (**a**) opened sensor half with eight arc-shaped (left) and eight ring-shaped (right) flow noise antennas (FNAs); (**b**) groove detail of one FNA; (**c**) longitudinal section with the electrode designations used in the text, and section A–A through the four segments a–d of arc electrode A_9_.

The sensor (Figure 6) carries eight ring electrodes (A_0_–A_7_) and eight arc electrodes (A_8_–A_15_), each arc electrode comprising four 60° segments (a–d) in one groove, separated by four equal 30° gaps that insulate adjacent segments and reduce their coupling. The FNAs are insulated square copper wires set in grooves; their dimensions and axial spacing are given in Figure 6b,c. Sixteen differential DAS channels were recorded: six rings (A_0_, A_1_, A_3_–A_6_) and the eight segments A_8_.a–d and A_9_.a–d through their SCCs, and two segments (A_10_.a, A_11_.a) connected directly to the DAS as integrity monitors for cable or electrode faults. Segment A_9_.c showed no response in any record and was excluded, leaving 13 conditioned channels (six rings and seven arc segments) for classification.

**Figure 7:**
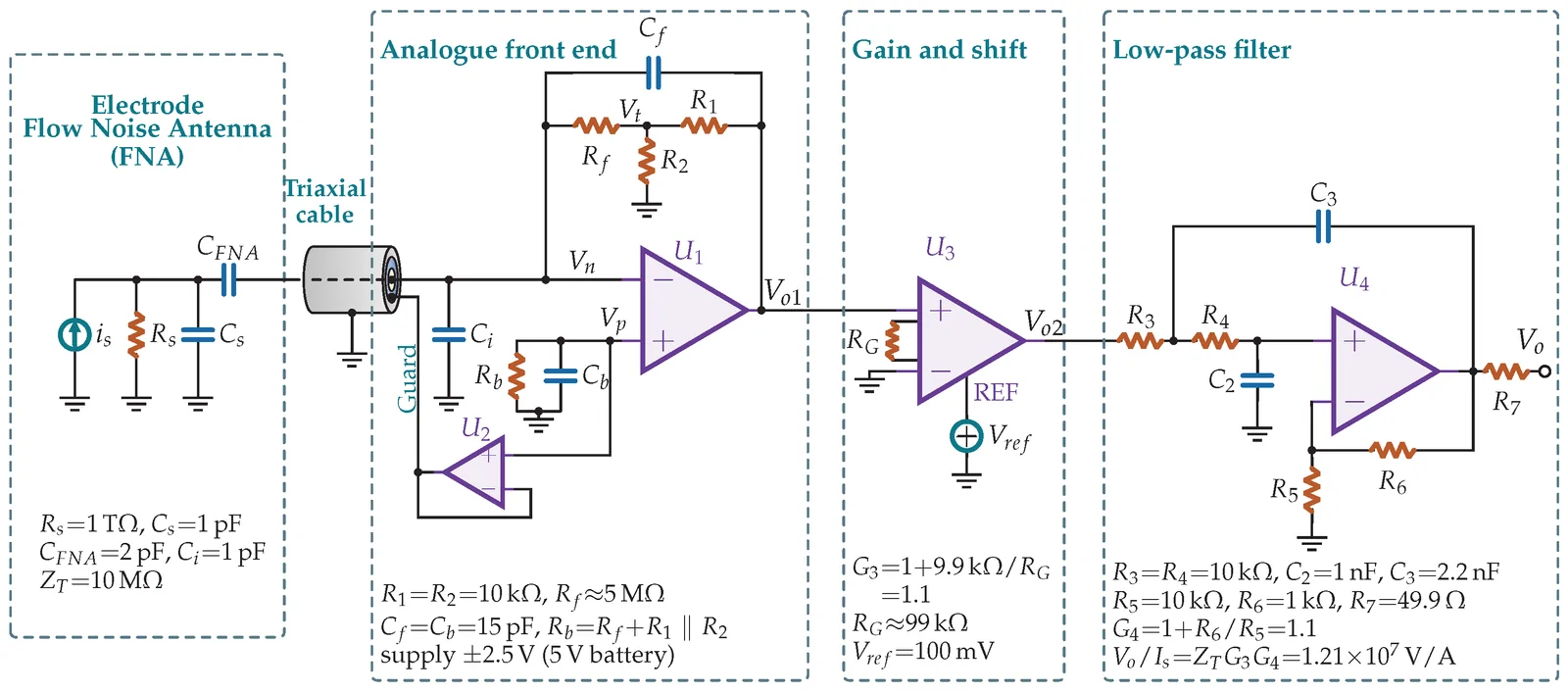
Schematic of one channel of the signal conditioning circuit: transimpedance amplifier (TIA) with guarded triaxial input, instrumentation amplifier and Sallen–Key low-pass filter. $C_{i}$: input capacitance at the U_1_ input, including the electrode-to-shield capacitance $C_{e}$ (Figure 2); $R_{b}$, $C_{b}$: bias-current compensation network.

**Figure A1:**
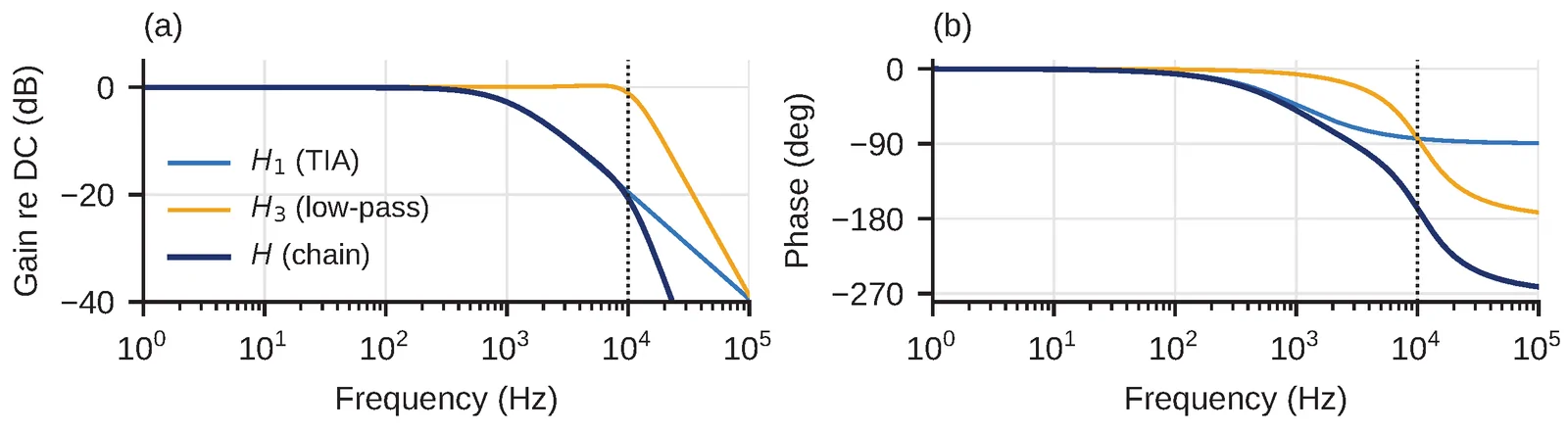
Frequency response of the conditioning chain computed from Equation (3) with ideal op-amps: (**a**) gain relative to the DC value and (**b**) phase of the front end $H_{1}$, the Sallen–Key low-pass filter $H_{3}$ and the chain *H* ($H_{2}=G_{3}$ is frequency independent; front-end sign inversion omitted); dotted line: Nyquist frequency of the acquisition (10 kHz).

As shown in Figure 7, each conditioned channel cascades three stages whose combined transfer function for ideal operational amplifiers (op-amps) is given by Equation (3). The AFE $H_{1}\left(s\right)$ is a transimpedance amplifier (TIA) around the electrometer-grade op-amp U_1_ (LMP7721 [34]), with an input bias current of a few femtoamperes, very high input impedance and low offset and drift; the guard buffer U_2_ (LMP7715 [35]) drives the inner shield of the triaxial input cable. A T-network in the feedback path of U_1_ realises the equivalent feedback resistance $Z_{T}=10$ MΩ with lower-value resistors, avoiding costly very-high-value resistors and their leakage and temperature sensitivity; its higher noise and offset gain were acceptable because the median no-flow output (Section 3.3) remained below the flow signals at all flow rates. The second stage $H_{2}$, the instrumentation amplifier U_3_ (AD8421 [36]), rejects common-mode interference and ground offsets, with gain $G_{3}=1+9.9\;k\Omega /R_{G}$ and an output level shift set by $V_{ref}=100$ mV. The Sallen–Key low-pass filter $H_{3}$ around U_4_ (ADA4077-1 [37]), of gain $G_{4}$, and the front-end pole together attenuate the signal by about 20 dB at the 10 kHz Nyquist frequency of the acquisition. With $G_{3}=G_{4}=1.1$ ($R_{G}\approx 99$ kΩ), the total transimpedance gain, set mainly by the T-network, is $10^{7}\times 1.1\times 1.1=1.21\times 10^{7}$ V/A, keeping the signals within the ±1 V DAS input range up to the highest flow rates that the air stream could convey without deposition and blockage; for weaker signals, $G_{3}$ can be raised to $10^{4}$ by changing $R_{G}$ alone. Resistors had a tolerance of at most 1% and a temperature coefficient of resistance of 25 ppm/°C, capacitors at most 5% and 30 ppm/°C.

(3) $$\begin{array}{cc} H(s) & =H_{1}\left(s\right)H_{2}\left(s\right)H_{3}\left(s\right)\\ \; & =-\frac{Z_{T}}{1+sZ_{T}C_{f}}\;G_{3}\;\frac{G_{4}}{1+s\left[\left(R_{3}+R_{4}\right)C_{2}+R_{3}C_{3}\left(1-G_{4}\right)\right]+s^{2}R_{3}R_{4}C_{2}C_{3}} \end{array}$$

where $H\left(s\right)=V_{o}/I_{s}$ is the overall transimpedance of the chain from the electrode current $I_{s}\left(s\right)$, the Laplace transform of $i_{s}\left(t\right)$, to the output voltage $V_{o}$; $H_{1}\left(s\right)=V_{o1}/I_{s}=-Z_{T}/\left(1+sZ_{T}C_{f}\right)$ is the front-end transimpedance, with the equivalent feedback resistance of the T-network $Z_{T}=R_{f}\left(1+R_{1}/R_{2}\right)+R_{1}$ and the feedback capacitance $C_{f}$; $H_{2}=V_{o2}/V_{o1}=G_{3}$ is the gain of the instrumentation amplifier and $H_{3}\left(s\right)=V_{o}/V_{o2}$ is the transfer function of the Sallen–Key low-pass filter with resistors $R_{3}$ and $R_{4}$, capacitors $C_{2}$ (to ground) and $C_{3}$ (feedback) and gain $G_{4}=1+R_{6}/R_{5}$ (Figure 7). With ideal op-amps, H(s) has no finite zeros and three poles: the real front-end pole at $f_{1}=1/\left(2\pi Z_{T}C_{f}\right)\approx 1.06$ kHz ($C_{f}=15$ pF), which sets the signal bandwidth, and the complex-conjugate filter pair at $f_{0}=1/\left(2\pi \sqrt{R_{3}R_{4}C_{2}C_{3}}\right)\approx 10.7$ kHz with quality factor $Q_{f}\approx 0.83$. The chain stays within 1 dB of its DC gain up to 0.5 kHz and lags by about 50° at 1 kHz, and the filter peaks by only 0.3 dB (Appendix A.1, Figure A1). Above about 0.5 kHz, the spectrum of the induced current, and with it the frequency-domain features 17–21 of Table 1, is, therefore, shaped by the front end as well as by the flow. Before the boards were built, each stage was checked with SPICE (LTspice version 26.1.1, manufacturers’ op-amp macromodels) for DC operating points, frequency response, loop stability, noise, transient response and robustness to component tolerances and temperature.

**Figure A2:**
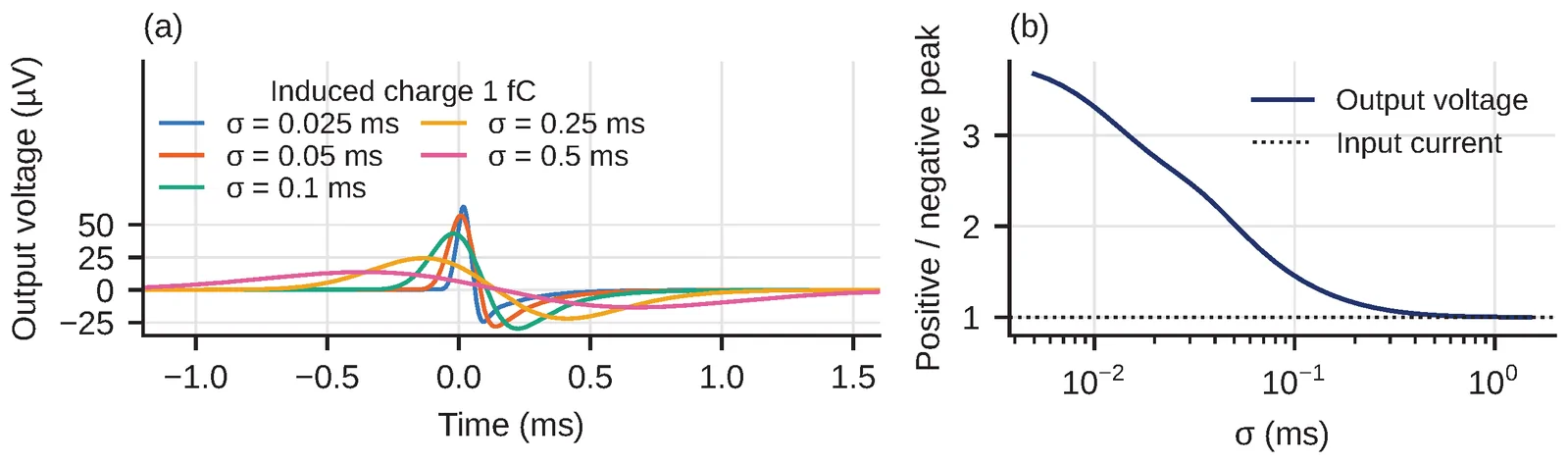
Response of the conditioning chain (Equation (3), ideal op-amps, without the front-end sign inversion) to the bipolar current induced by a particle, modelled as the time derivative of a Gaussian-induced charge of 1 fC with standard deviation σ: (**a**) output voltage for five values of σ; (**b**) ratio of the positive to the negative output peak versus σ.

The front end distorts short pulses (Figure A2, Appendix A.1: response to the bipolar current i=dq/dt of a passing particle, modelled as a Gaussian induced charge of width σ, so that the two current peaks are equal). For σ of 0.25 ms and more, the two output lobes remain nearly symmetric (peak ratio at most 1.1); shorter pulses are stretched by the 0.15 ms time constant of the front-end pole into a large positive and a smaller, slower negative lobe (peak ratio 2.0 at σ=0.05 ms). The positive peak, at most about 64 μV per fC, then measures the charge rather than the peak current. For σ below about 0.1 ms, the zero crossings, waveform length, slope-sign changes and mean frequency are, therefore, limited by the front end, and the skewness, zero for the symmetric input, acquires a sign set by the polarity of the induced charge.

The SCC was powered from a 5 V battery split into ±2.5 V rails to minimise supply-borne noise. The assembled boards were calibrated with currents from a Keithley 6221 current source, measured with a Keithley 6517B electrometer on its 20 pA to 2 nA ranges (accuracy on the 20 pA range ±(1% of reading + 3 fA)). A 16-bit National Instruments (NI) USB-6259 DAS digitised the channels in differential mode at 20 kS/s per channel with an input range of ±1 V (Figure 1); the data were logged with NI SignalExpress and inspected for consistency with NI FlexLogger.

### 3.3. Dataset and Signal Characteristics

**Figure 8:**
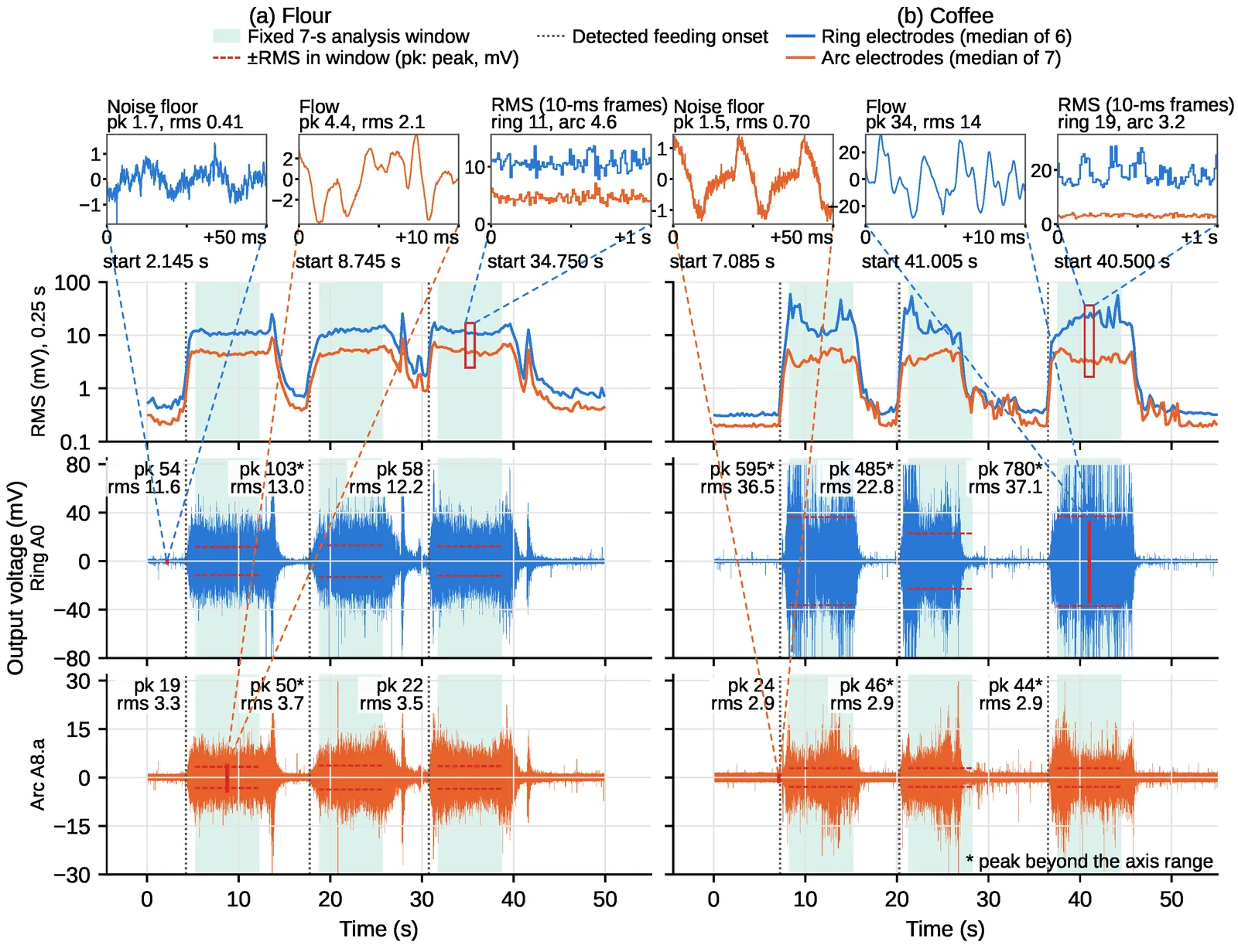
Example records at 15 m/s and 10 g/s: (**a**) flour; (**b**) coffee. Top row: median root-mean-square (RMS) values of the ring and of the arc electrodes; lower rows: raw output as min/max envelopes, on a common vertical scale for both materials. Insets, whose sources are marked by red boxes, give values in mV: noise floor, the 50 ms segment before the first onset in which the 50 Hz mains component is most regular; flow, 10 ms at a plateau centre, resolving individual particle pulses (flour, first plateau; coffee, last plateau); RMS over 10 ms frames in the central (fourth) 1 s analysis block of the last plateau.

Only the established-flow part of each feeding cycle was employed. Feeding onsets were detected from the RMS of each channel over non-overlapping 0.25 s frames, as the first frame after a no-flow interval at which the median over the 13 conditioned channels exceeded a threshold set midway, on a logarithmic scale, between the 10th and 90th percentiles of this median trace. A fixed window from 1 s to 8 s after each onset, which excludes the feeder start-up transient and ends before the nominal 10 s feeding period (Figure 8), was divided into seven non-overlapping 1 s blocks of 20,000 samples per channel, common to all channels. In 18 of the 117 feeding cycles, mainly at the lowest flow rates, the batch was discharged in less than 8 s, so that the actual rate exceeded the nominal $Q_{m}$ used as the class label; blocks whose median RMS fell below 40% of the median of their window were, therefore, discarded as no-flow blocks (21 of 819). The remaining 391 flour blocks (19 TCs) and 407 coffee blocks (20 TCs), 61–84 per class and each tagged with material, air velocity, feeding cycle and class, form the dataset; the features of Table 1 were extracted from every block.

Each feeding cycle produces a plateau whose RMS level exceeds the no-flow level by a factor of about 2 to 40 (Table 3), with ring electrodes responding more strongly than arc electrodes (median RMS ratio 2.8; Figure 8). Without particles, the median RMS of the 13 channels was 0.22–0.54 mV, i.e., about 18–45 pA of input current (Table 3); this noise floor was similar for all channels except arc segment A_8_.c (1.4–2.6 mV). In the example records, ring A_0_ gave 11.6–13.0 mV for flour and 22.8–37.1 mV for coffee, about 29–30 and 30–35 dB above the noise floor of the insets. The materials differ mainly in the impulsive content of the ring signals: peaks of 54–103 mV for flour but 485–780 mV for coffee raised the peak-to-RMS ratio from 5–8 to 16–21, whereas arc A_8_.a gave about 3 mV RMS with peaks of 19–50 mV for both. The flour plateaus were flat and the coffee plateaus fluctuated more; in the second coffee cycle, the feeding ended before the window closed (blocks removed by the 40% criterion). Over all records, the median RMS rose with the mass flow rate from 1.1–2.3 mV at 0.1 g/s to 3.1–9.4 mV at 10 g/s (roughly 0.1 to 0.8 nA; Table 3). The slope, however, depended on velocity and material: at 30 m/s, most flour levels were 15–55% lower than at 15–25 m/s, and the coffee levels varied only between 1.7 and 3.1 mV across the five classes, with 5 g/s below 1 g/s. The same level can, therefore, correspond to different mass flow rates at different velocities, as expected from Equation (1).

**Figure 9:**
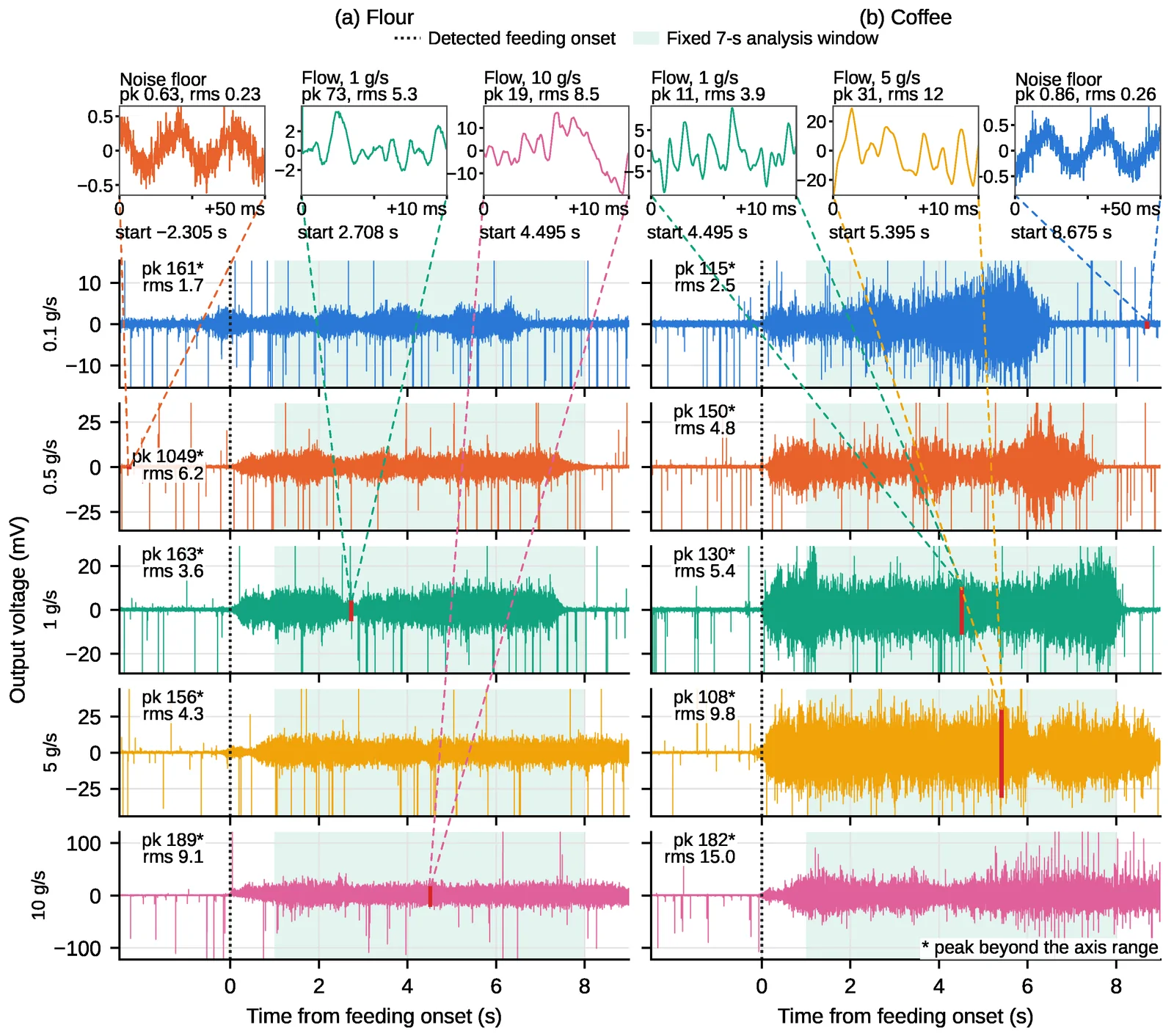
Ring electrode A_0_, first feeding cycle at 30 m/s: (**a**) flour; (**b**) coffee. Line colours distinguish the five mass flow rates (rows). Both materials share the vertical scale at each rate; impulsive events beyond it are truncated. pk, rms: peak and RMS values (mV) in the analysis window, left of each onset line, and in the insets, whose sources are marked by red boxes. Noise floor: dominated by 50 Hz mains, flour before the onset, coffee after the end of the feeding cycle. Flow: individual particle pulses from the largest peak between 2 and 3 s (flour, 1 g/s), the plateau centre (flour, 10 g/s; coffee, 1 g/s) and the strongest flow (coffee, 5 g/s).

**Figure 10:**
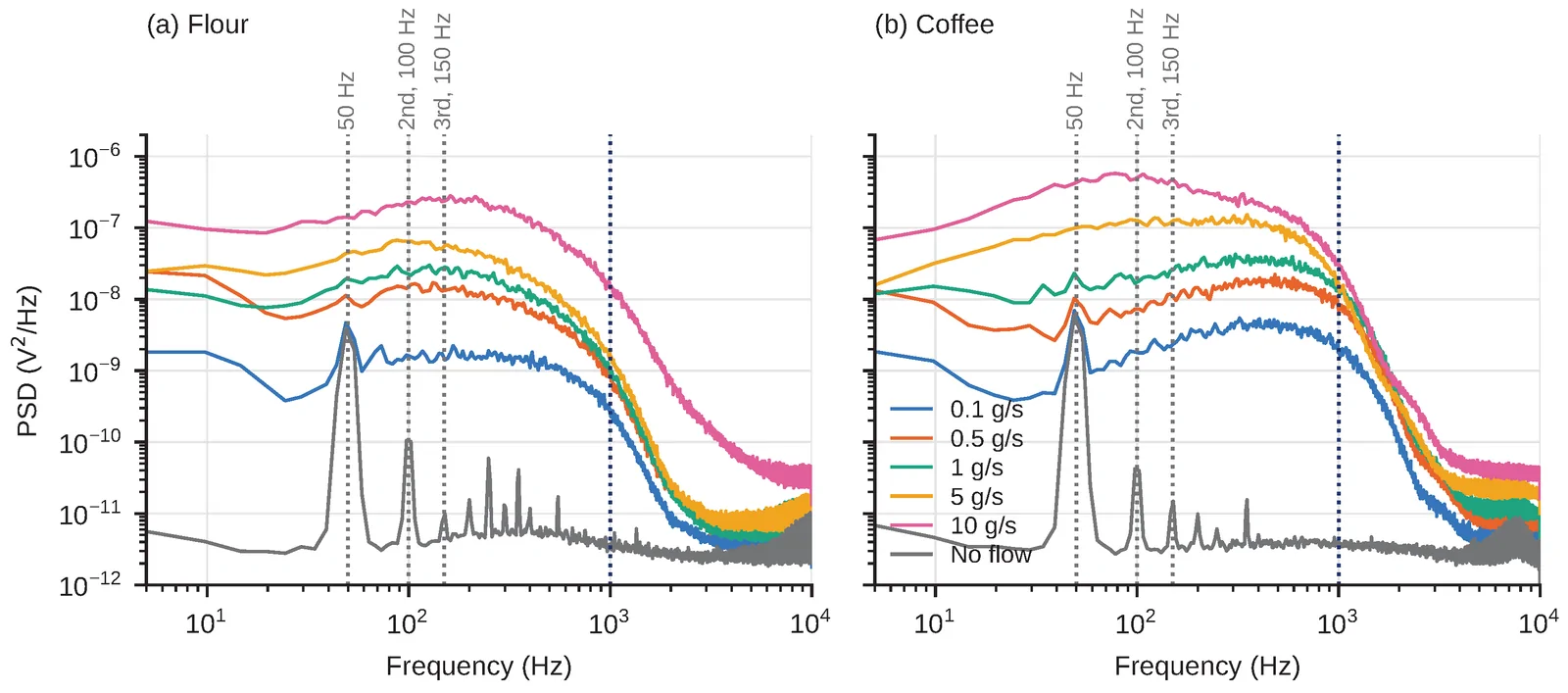
Power spectral density of ring electrode A_0_ at 30 m/s: (**a**) flour; (**b**) coffee. Welch estimate (4096-point segments) after ±3σ clipping, averaged over the three fixed analysis windows of each test condition; no flow: pooled intervals before the first feeding onset of the same five records; dotted lines: 1 kHz front-end pole (navy) and 50 Hz mains and its harmonics.

At 30 m/s, the output of ring A_0_ (Figure 9) rises with the mass flow rate. After the ±3σ clipping, its RMS in the analysis window, averaged over the three feeding cycles, increases from about 1 to 10 mV between 0.1 and 10 g/s for flour and from about 2 to 14 mV for coffee and, unlike the median over all channels (Table 3), is higher for coffee at every rate. Short impulsive events, superimposed on the broadband fluctuation at all rates, are stronger for coffee (99.9th-percentile amplitude about 95 mV at 10 g/s, against 43 mV for flour) and limited by clipping, which removes 0.3–1.5% of the samples of these records. The power spectral densities (Figure 10a,b) generally rise with the mass flow rate and remain nearly flat up to about 1 kHz, where they roll off at the front-end pole. The materials differ in spectral shape: for coffee, the power at 5–10 g/s is concentrated between 100 and 500 Hz, whereas the flour spectra at 0.5–5 g/s are poorly separated. The frequency-domain features 17–21 of Table 1 summarise the spectrum of each block: at 30 m/s, the median mean frequency (MNF) of ring A_0_ falls from about 670 to 400 Hz between 1 and 10 g/s for coffee, whereas for flour it lies between 300 and 390 Hz at 0.5–10 g/s and reaches 650 Hz only at 0.1 g/s. Without flow (grey), the spectrum consists of the 50 Hz mains component and its harmonics on a floor of about 2–$5\times 10^{-12}$ V^2^/Hz and lies 23 dB (flour) and 30 dB (coffee) below the 0.1 g/s spectrum between 100 Hz and 1 kHz; the spectra at the lowest rates approach this floor above about 2.5–4 kHz. The 50 Hz component is clearest at 0.1 g/s and fades as the flow signal grows.

**Figure A3:**
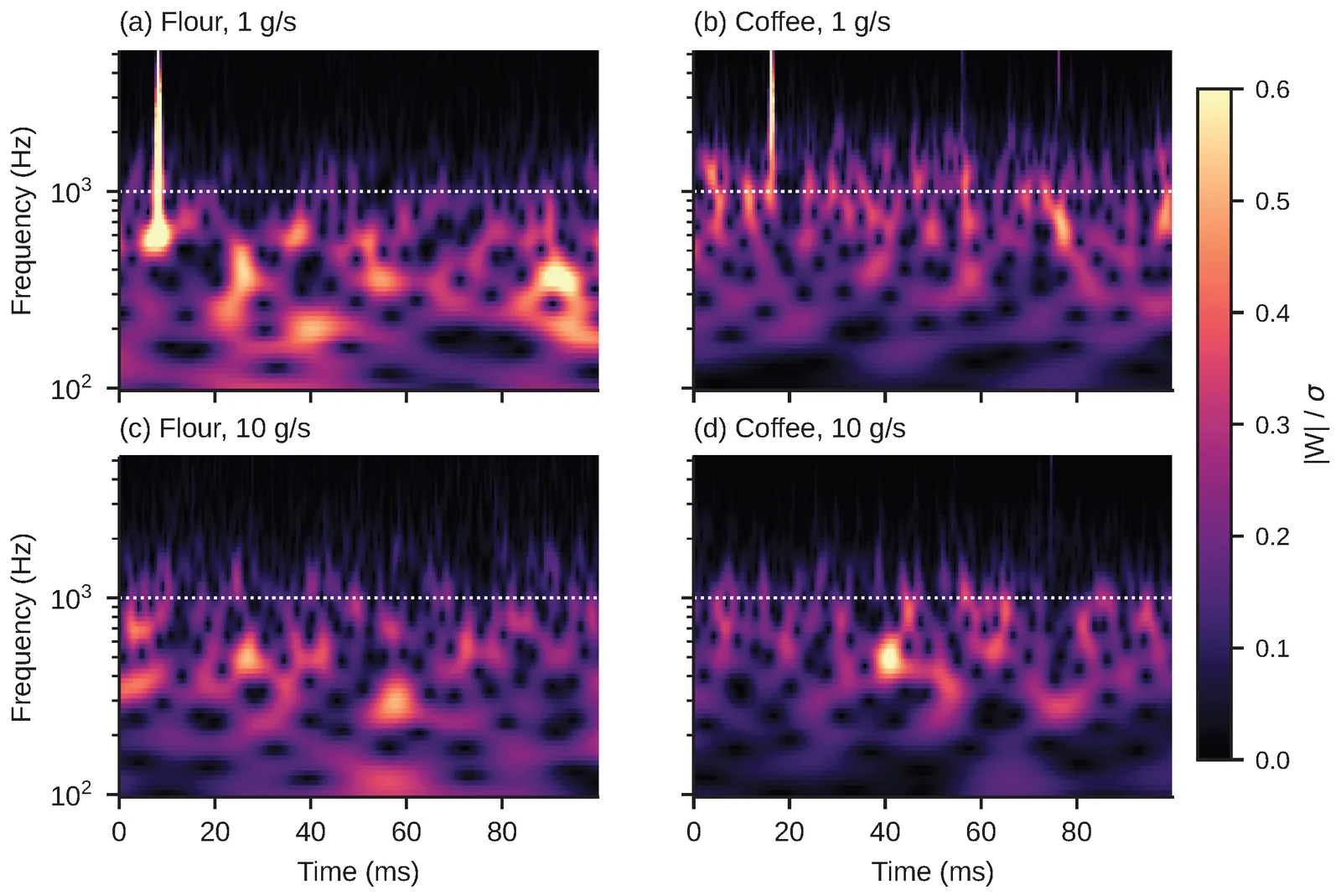
Morlet wavelet scalograms ($\omega_{0}=6$) of ring electrode A_0_ at 30 m/s over 100 ms at the centre of the first fixed analysis window: (**a**) flour, 1 g/s; (**b**) coffee, 1 g/s; (**c**) flour, 10 g/s; (**d**) coffee, 10 g/s. Unclipped signal divided by the standard deviation σ of its 7 s window; common colour scale; dotted line: 1 kHz front-end pole; corresponding spectra in Figure 10.

**Figure A4:**
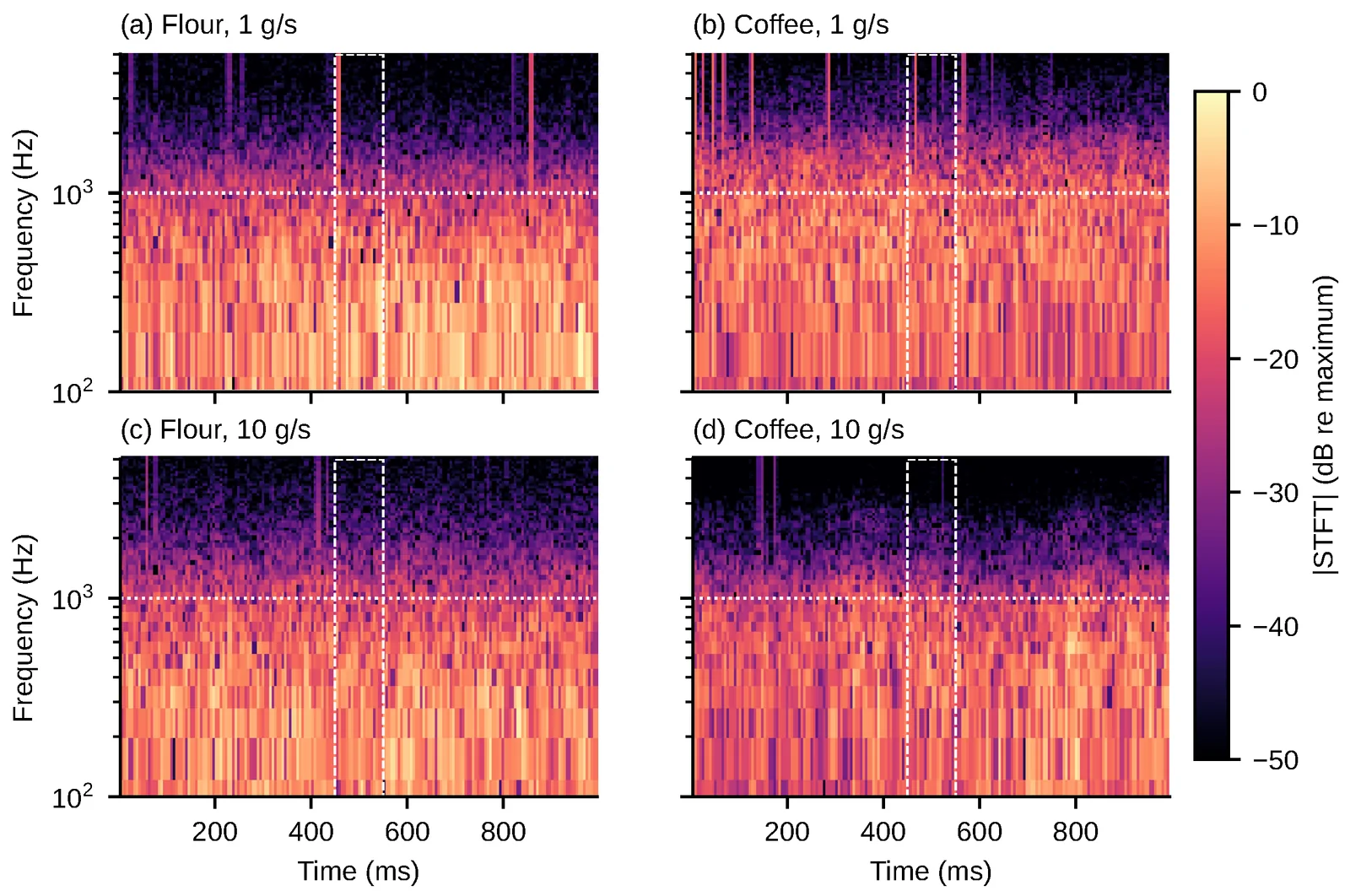
Short-time Fourier transform (STFT) magnitude of ring electrode A_0_ at 30 m/s over the 1 s block centred on the first fixed analysis window, computed as for the time–frequency features (Hann window of 256 samples, 12.8 ms, 50% overlap); panels and normalisation as in Figure A3 (dashed box: its 100 ms segment); colour scale in dB relative to the maximum of the four panels; dotted line: 1 kHz front-end pole.

**Figure A5:**
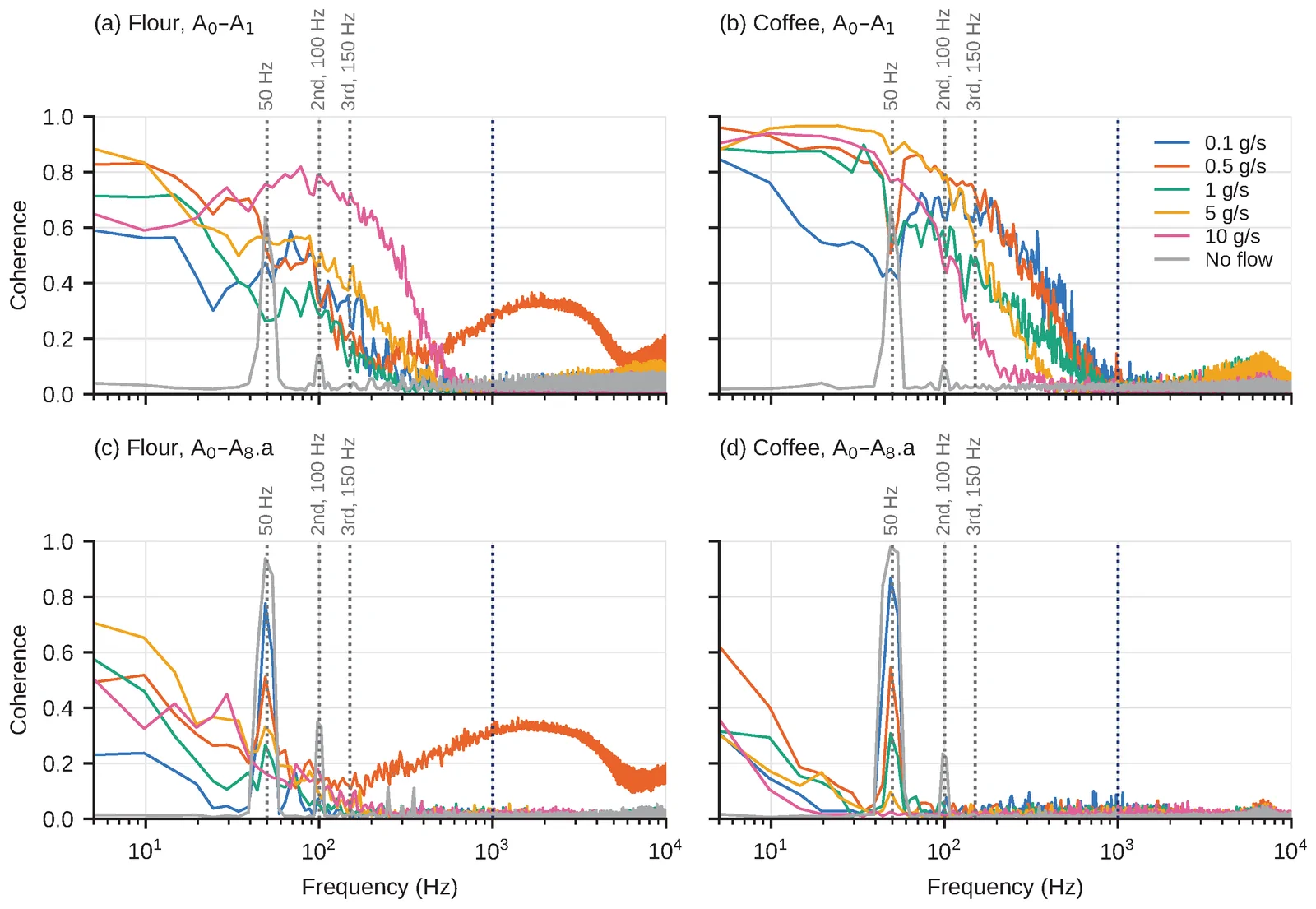
Magnitude-squared coherence of the unclipped signals at 30 m/s for the five mass flow rates between ring electrode A_0_ and (**a**,**b**) the adjacent ring A_1_ or (**c**,**d**) arc segment A_8_.a: (**a**,**c**) flour; (**b**,**d**) coffee. Welch estimate (4096-point segments) averaged over the three fixed analysis windows of each test condition; no flow: pooled intervals before the first feeding onset of the same five records; dotted lines: 1 kHz front-end pole (navy) and 50 Hz mains frequency and its harmonics.

Morlet wavelet scalograms of 100 ms segments of ring A_0_ and the STFT magnitude of the enclosing 1 s block (Figure A3 and Figure A4, Appendix A.2) show the time–frequency structure behind these spectra, and Figure A5 shows the coherence between neighbouring electrodes, including that of the 50 Hz component. At 1 g/s, the fluctuation occupies 100 Hz to 1 kHz unevenly in time, mainly below about 600 Hz for flour and between about 500 Hz and 1 kHz for coffee, and individual pulses appear as vertical streaks extending beyond the 1 kHz pole; at 10 g/s, the pulses overlap into a nearly continuous band and little energy remains above the pole. Owing to the ±3σ clipping before feature extraction, the kurtosis and the peak-based features 8–11 describe the amplitude distribution within ±3σ. The mean-crossing irregularity reflects mainly the concentration of the energy in frequency rather than its unevenness in time: in these records, the coefficient of variation of the STFT magnitude across frequency bins (1.5–2.6) exceeds that of its temporal envelope (0.2–0.4) severalfold.

**Figure A6:**
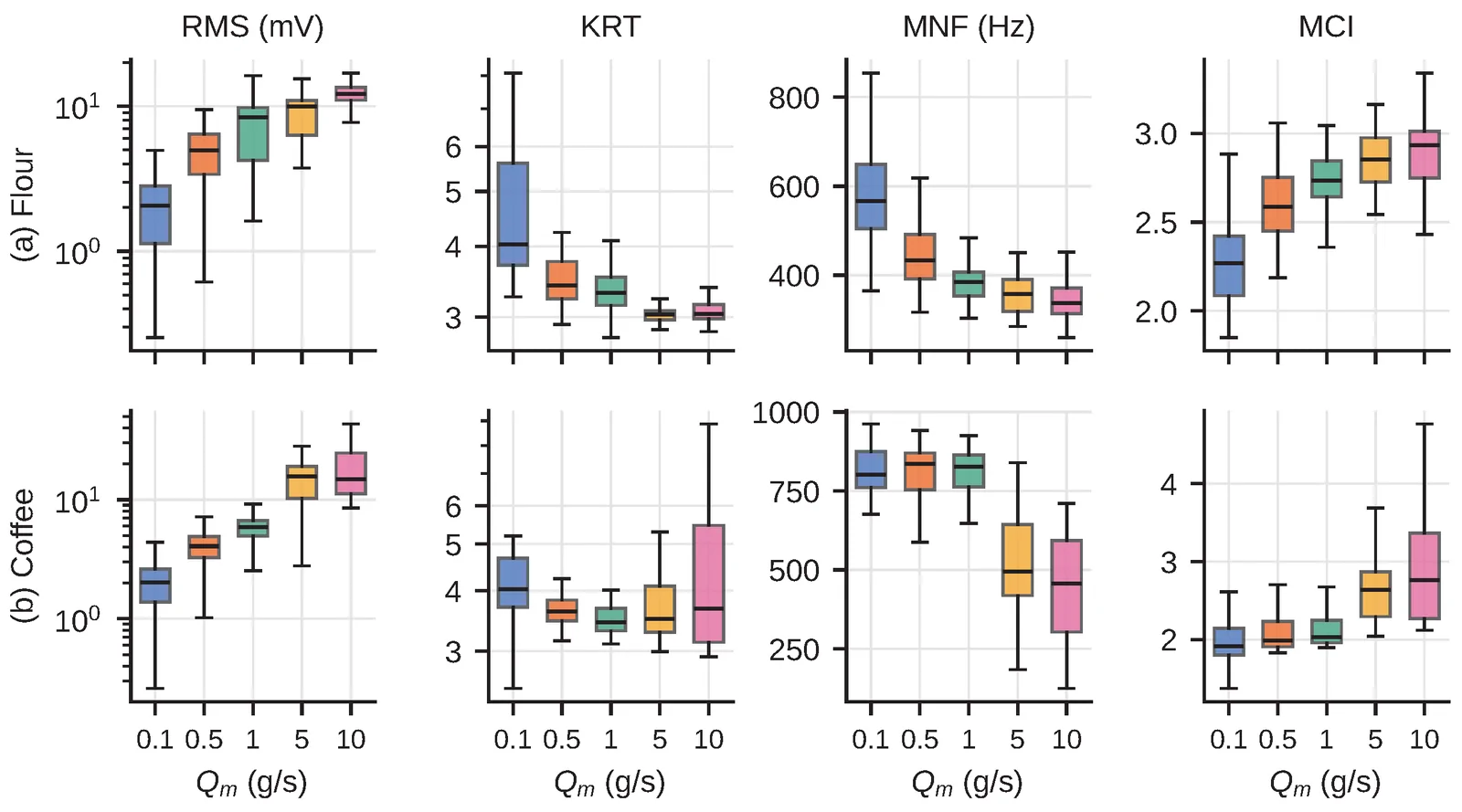
Four features of Table 1 per mass flow rate class over the 1 s blocks of ring electrode A_0_, all air velocities and feeding cycles pooled: (**a**) flour; (**b**) coffee. Columns: RMS, kurtosis KRT, mean frequency MNF and mean-crossing irregularity MCI; RMS and KRT on logarithmic axes. Boxes: median and interquartile range; whiskers: 1.5 times the interquartile range, beyond which points are omitted for readability.

In Figure A6 (Appendix A.3; ring A_0_, all velocities and feeding cycles pooled), four features from the three domains of Table 1 change with class, but neighbouring classes overlap. The RMS rises with the mass flow rate for both materials; the mean frequency falls with it for flour (median 566 to 337 Hz) and, for coffee, only above 1 g/s (about 830 to 460 Hz); the kurtosis is highest at 0.1 g/s, where the pulses overlap least and the mean-crossing irregularity increases with the mass flow rate. For flour, the interquartile ranges of 0.5 and 1 g/s overlap in all four features; for coffee, those of 0.5 and 1 g/s overlap in all but the RMS, and those of 5 and 10 g/s in all four.

### 3.4. Classification Performance

**Figure 11:**
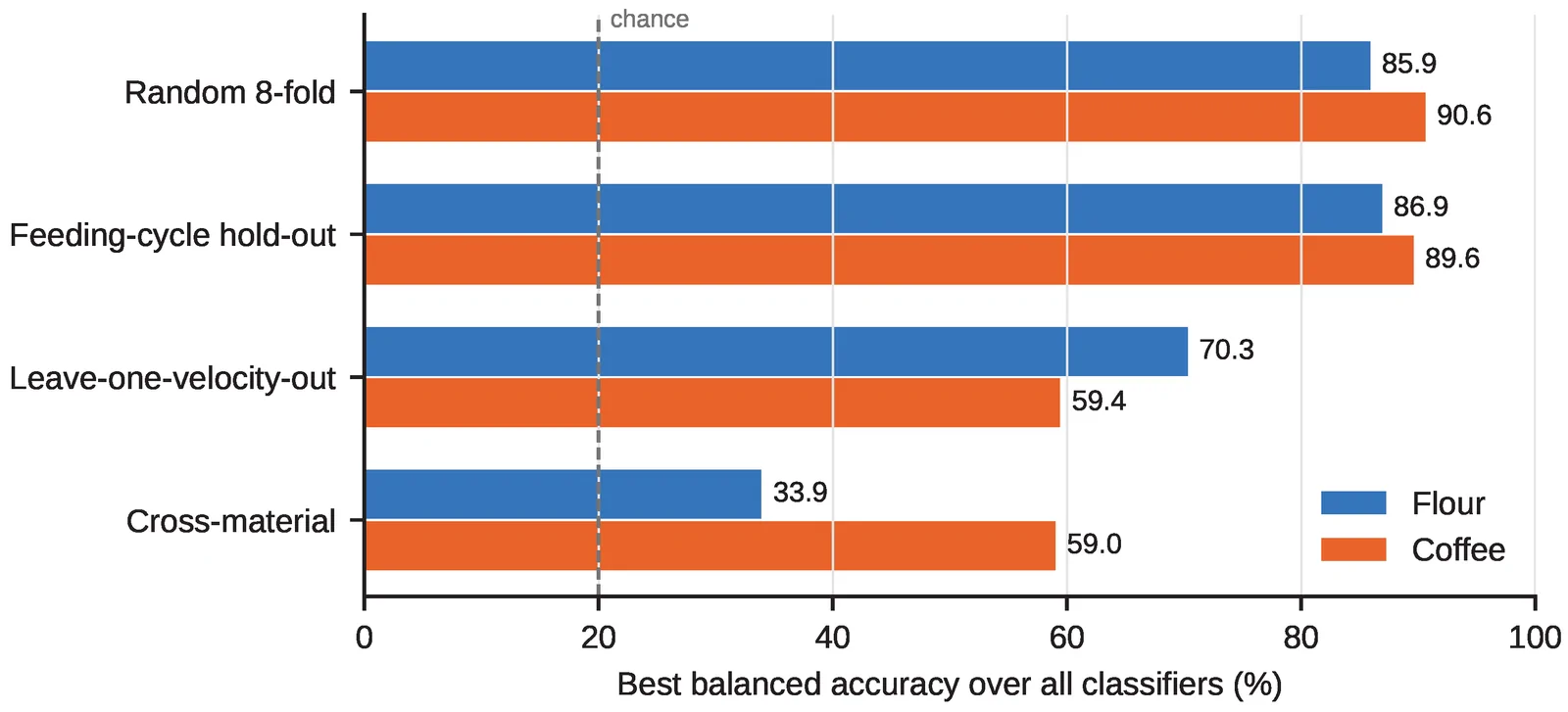
Best balanced accuracy over all classifiers for the three validation protocols and for cross-material testing (train on one material, test on the other; bars labelled by the training material).

Holding out complete feeding cycles changed the balanced accuracy only slightly (Table 4): the best models reached 86–91% with random 8-fold cross-validation and, under feeding-cycle hold-out, the best conventional classifiers achieved 85.4% (RBF-SVM) and 84.2 ± 0.3% (MLP) for flour and 88.1 ± 1.2% (MLP) and 85.5% (RBF-SVM) for coffee. Random splitting thus inflated the balanced accuracy of the best feature-based models by only 0.5–3 points, because the analysis windows exclude the start-up transients and because feeding-cycle hold-out also draws training and test blocks from the same records (Section 3.6). Under leave-one-velocity-out, by contrast, the balanced accuracy fell to 47–70% across classifiers (Table 4; best values in Figure 11). The standard deviation over five seeds was at most 1.4 points for MLP and RF, so the differences between the best models are comparable to the run-to-run variation.

The best deep models exceeded the best conventional classifiers by only 1–2 points; learned and engineered features performed comparably, and no approach was superior for both materials. Among the conventional classifiers (CVA, SVMs, MLP and RF), all but CVA performed within a few points of one another under 8-fold cross-validation and feeding-cycle hold-out; CVA was the weakest for flour (80.1% under feeding-cycle hold-out) but close to the best models for coffee (85.3%), for which the linear SVM was the weakest (81.8%). In the preliminary analysis of these data [11], the feature matrix of each class had been divided into 16×14 sub-images, resized to 224×224 and classified with a custom 2D CNN and with ImageNet-pretrained ResNet-18, ResNet-34 and ResNet-50 networks; the highest accuracy, 93.6% with ResNet-34, was obtained with random 70/10/20 splitting. Re-evaluated under the leakage-controlled validation protocols (Table 4), the CNNs trained from scratch on the 13×29 electrode-by-feature maps, with or without resizing, reached 76.5–80.7% for flour and 77.0–84.9% for coffee under feeding-cycle hold-out, so arranging the features as a map gave no advantage over a flat vector, as expected for about 400 blocks per material. ImageNet pretraining may partly compensate for the small dataset: the fine-tuned ResNet-34 gave the highest coffee balanced accuracy (89.6%, with 97.8% of predictions within one class), about 1.5 points above the MLP, but it did not do so for flour (82.9%). The end-to-end CNN on the raw signals gave the highest flour balanced accuracy (86.9%), with all predictions within one class, but was about six points below the MLP for coffee (82.2%).

**Figure 12:**
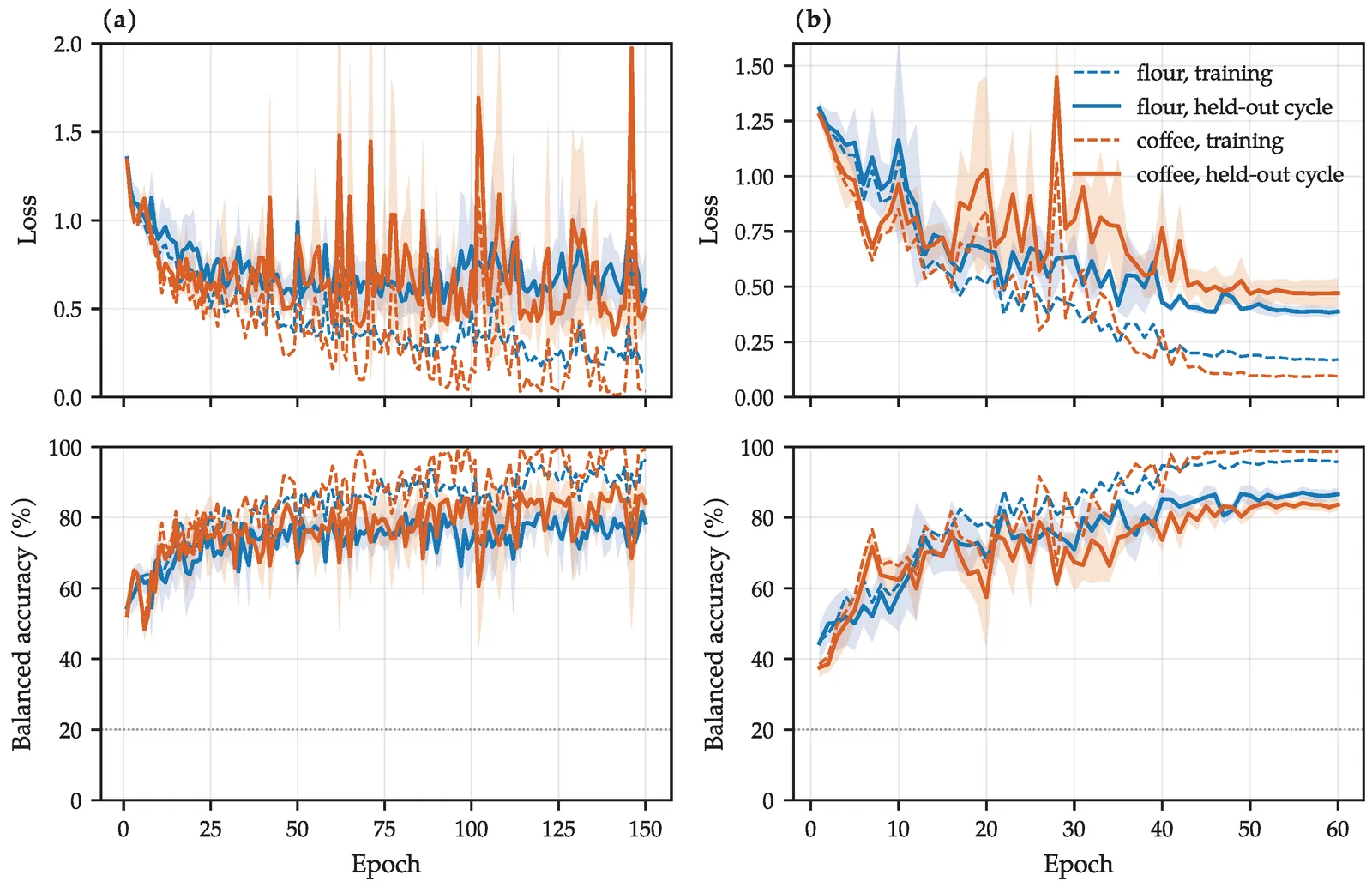
Learning curves under feeding-cycle hold-out (one seed; lines: mean over the three folds; shading: ±1 standard deviation of the held-out cycle): (**a**) feature-based 2D CNN (150 epochs, constant learning rate); (**b**) end-to-end CNN on the raw signals (60 epochs, cosine learning-rate schedule). Upper row: class-weighted cross-entropy loss; lower row: balanced accuracy (dotted line: chance level).

**Figure A7:**
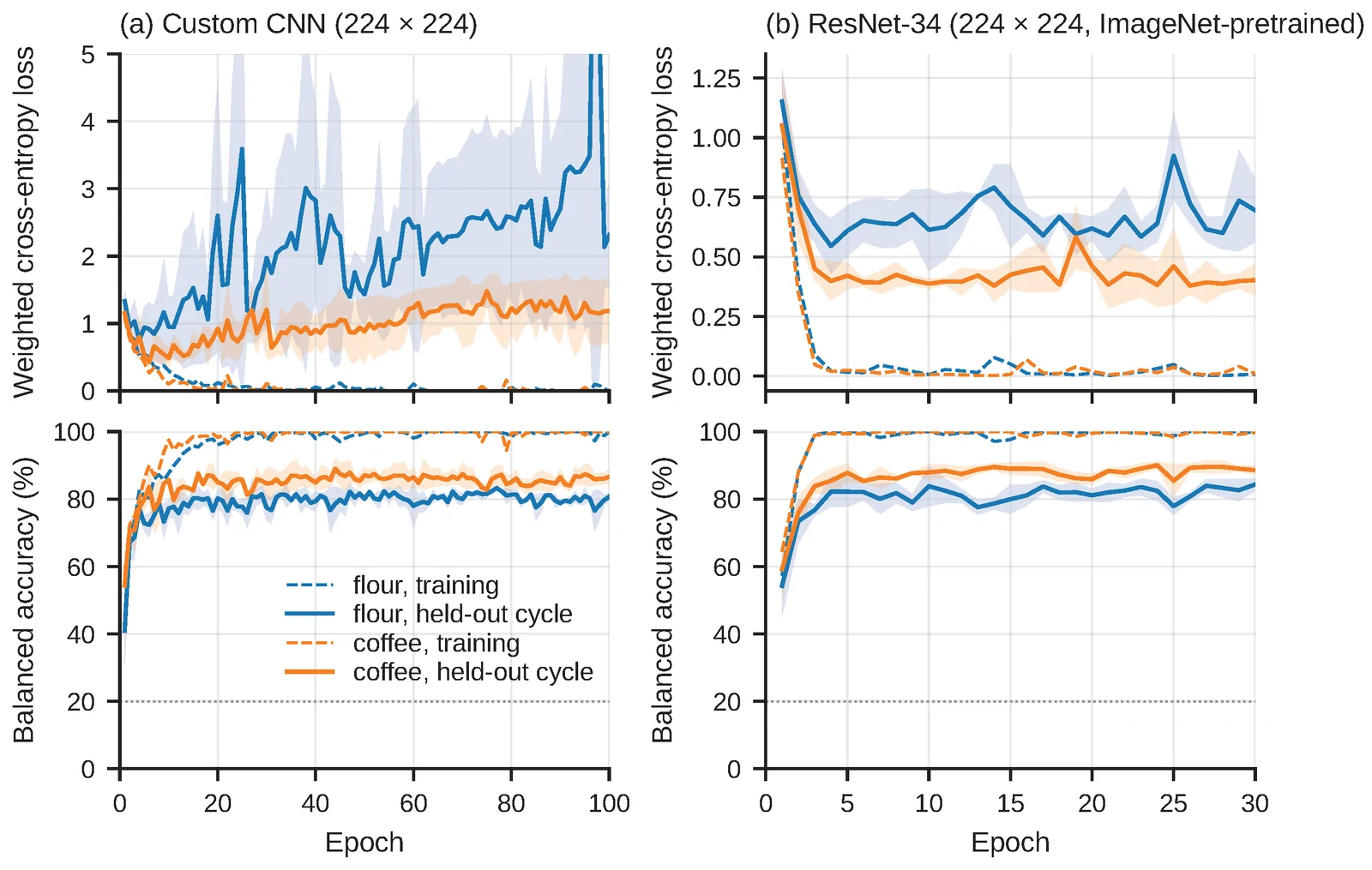
Learning curves of the 224×224 image networks under feeding-cycle hold-out (one seed; batch size 16; lines: mean over the three folds; shading: ±1 standard deviation of the held-out cycle): (**a**) custom CNN on the 13×29 electrode-by-feature map resized to 224×224 (100 epochs, learning rate $10^{-3}$; loss axis truncated at 5); (**b**) ImageNet-pretrained ResNet-34 with all layers fine-tuned (30 epochs, learning rate $10^{-4}$). Upper row: class-weighted cross-entropy loss; lower row: balanced accuracy (dotted line: chance level).

Under feeding-cycle hold-out, the learning curves of the feature-based 2D CNN and the end-to-end CNN (Figure 12, one seed) show moderate overfitting to the training cycles but no loss of held-out accuracy. The training loss decreased steadily, whereas the held-out loss levelled off after about 40 epochs for the 2D CNN and 45–50 epochs for the end-to-end CNN. The held-out balanced accuracy did not decrease towards the end of training (2D CNN: 78.6% for flour and 84.1% for coffee; end-to-end CNN: 86.6% and 83.6%; Table 4 gives the means over all seeds) but stayed 9–17 points below the training accuracy. The fluctuations of the 2D CNN are consistent with its constant learning rate, whereas the end-to-end CNN converges smoothly with its cosine learning-rate schedule. The learning curves of the two 224×224 image networks are given in Appendix A.3 (Figure A7).

**Figure 13:**
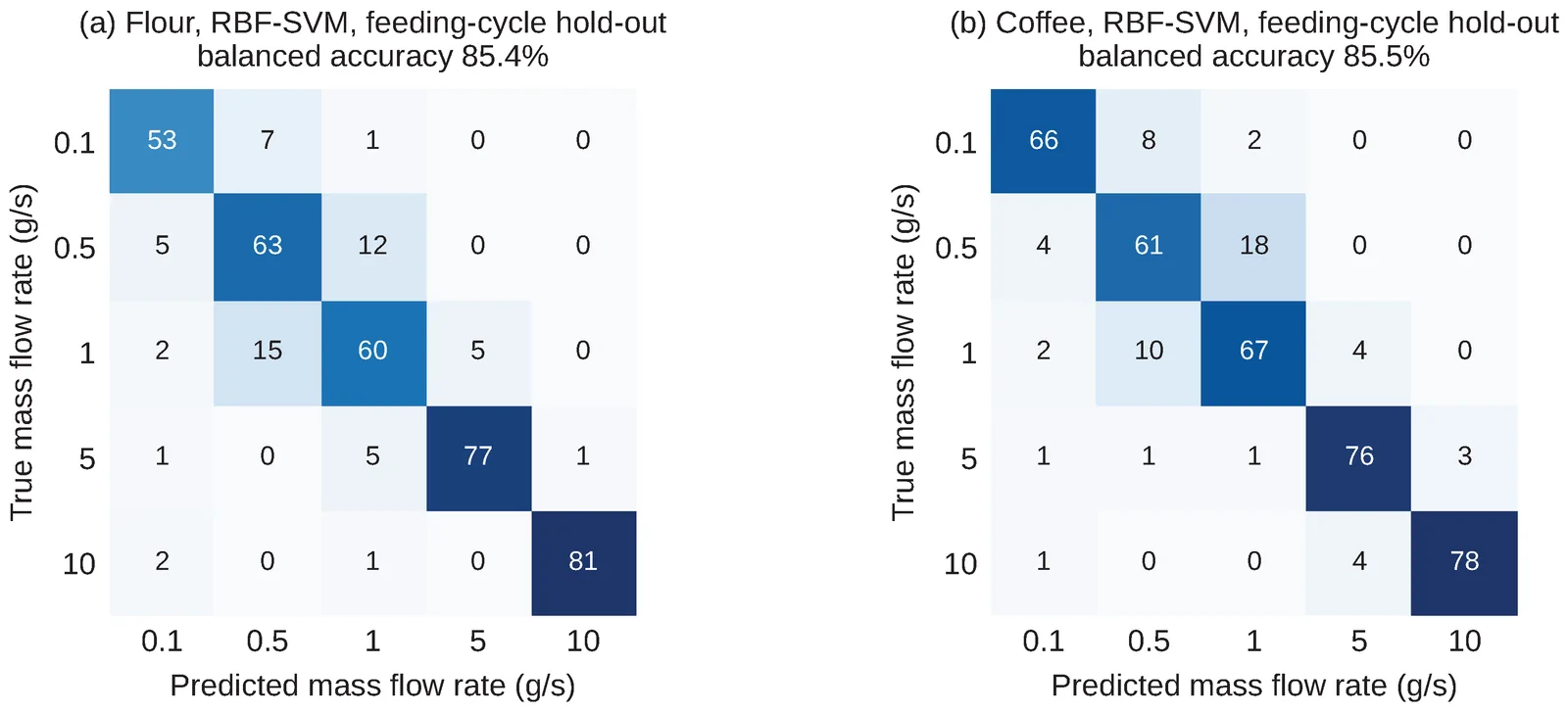
Confusion matrices of the RBF-SVM under feeding-cycle hold-out for (**a**) flour and (**b**) coffee.

Errors occur mainly between adjacent classes (Table 5, Figure 13): under feeding-cycle hold-out, 98.2% (flour) and 98.3% (coffee) of the RBF-SVM predictions were within one class of the true class. For both materials, the most frequent confusion was between 0.5 and 1 g/s (F1 scores of 74.5–78.4%), whereas the 0.1, 5 and 10 g/s classes reached 85.5–97.6%. The 0.5 and 1 g/s classes are those whose signal levels overlap most (Table 3) and whose feature distributions overlap for both materials (Appendix A.3, Figure A6); for coffee, their levels differ by less than 10% at 20 and 25 m/s. These errors cannot be attributed to a specific flow regime with the present data; possible physical causes of the overlap are discussed in Section 4.

### 3.5. Electrode Type and Generalisation Across Velocity and Material

**Figure A8:**
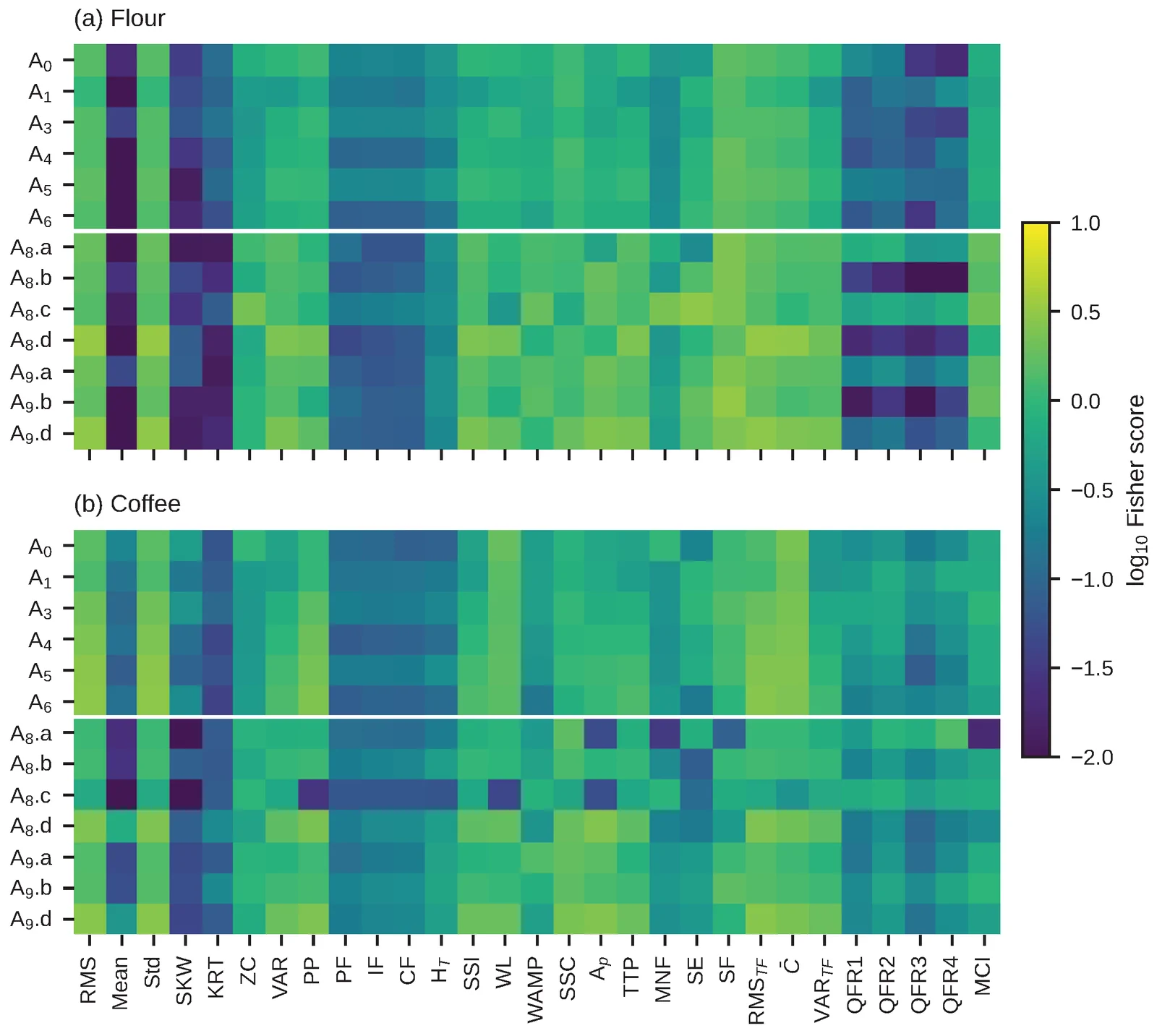
Fisher score of the 29 features of Table 1 (columns) for the 13 conditioned channels (rows, named as in Section 3.2), all air velocities and feeding cycles pooled: (**a**) flour; (**b**) coffee. Score: class-size-weighted ratio of the between-class to the within-class variance; horizontal line: boundary between the ring channels and the arc segments.

Each electrode type carries most of the discriminative information on its own (RBF-SVM on six ring, seven arc or all 13 channels; Table 6). Under feeding-cycle hold-out, the ring and arc subsets reached 78.9% and 82.8% for flour and 75.0% and 87.5% for coffee, against 85.4% and 85.5% for the combined set; under leave-one-velocity-out, the rings generalised better than the arcs, by 5 points for flour and 17 points for coffee. The per-channel Fisher scores of the 29 features of Table 1 (Appendix A.3) show the same pattern: within each air velocity, the arc channels scored higher than the rings for both materials, except coffee at 30 m/s. Pooling the velocities (Figure A8) lowered the scores of both types severalfold and, for coffee, brought the arcs below the rings (mean 0.77 against 0.81; flour: 1.05 against 0.63), consistent with their larger velocity dependence. The amplitude features 1, 3, 22 and 23 scored highest and the mean, skewness and kurtosis were below 0.2; arc segment A_8_.c scored lowest for coffee. For coffee, the combined set was below the arc subset under feeding-cycle hold-out and below the ring subset under leave-one-velocity-out, so a selection of channels may generalise better than the full set. The arc segments, which resolve the circumferential distribution, were more informative within the trained velocities, particularly for the coarser coffee, whereas the rings, which integrate over the cross-section, were less sensitive to a change in air velocity; their combination gave the best result for flour under both validation protocols.

The features of Table 1 are computed channel by channel and carry no information on the relation between channels. In the signal band from 100 Hz to 1 kHz, the magnitude-squared coherence between ring A_0_ and the adjacent ring A_1_ and between A_0_ and arc segment A_8_.a is below 0.3 for both pairs in all ten records (Figure A5, Appendix A.2; records of Figure 9 and Figure 10). These low values indicate weak linear coupling in this band, not statistical independence: the channels are not linearly redundant, and the 377-dimensional feature vector retains channel-specific information. Between 10 and 100 Hz, the two rings are coherent (0.4–0.9), whereas the ring–arc coherence is low (0.02–0.27) apart from the 50 Hz mains component; the coherence without flow and above the front-end pole is described in Appendix A.2.

The drop in accuracy under leave-one-velocity-out reflects the velocity dependence of the signal levels (Section 3.3). The largest errors occurred with 30 m/s held out (balanced accuracy 38–45% for the RBF-SVM and RF), which requires extrapolation to the velocity with the lowest signal levels and, for coffee, the narrowest spread between classes. Training on both materials together gave a similar leave-one-velocity-out accuracy (RF: 62.5%).

The method is not specific to one powder: with the same features and models, coffee was classified at least as well as flour (89.6% versus 86.9% under feeding-cycle hold-out). Models trained on one material, however, did not transfer reliably to the other: training on flour and testing on coffee gave 26–34% balanced accuracy, and the reverse 29–59%, although 59–94% of the predictions remained within one class. Because the two powders are expected to charge differently (Section 3.1), the absolute signal level for a given $Q_{m}$ differs between them, so a material-specific calibration is required, as also found for acoustic-emission models [24].

### 3.6. Regression, Recording-Order Control and Computational Cost

The same features also support an approximate continuous estimate, but on these data classification was more accurate than rounded regression. The SVR and RFR (Section 2.3) used the same features and splits, and their predictions were rounded to the nearest class. Under feeding-cycle hold-out, the RFR reached a mean absolute error of 0.127 (flour) and 0.116 (coffee) in $\log_{10}$ units, i.e., a typical factor of 1.34 and 1.31 between estimated and nominal flow rate. After rounding, it gave 79.2% and 82.1% balanced accuracy, about 8 points below the best classifiers, and under leave-one-velocity-out its error increased to 0.28 and 0.23. To test estimation between set-points, each intermediate flow rate was in turn withheld completely from training. The SVR then placed it between its neighbours: at true values of 0.5, 1 and 5 g/s, the median estimates were 0.59, 1.02 and 1.80 g/s for flour and 0.36, 0.53 and 3.74 g/s for coffee (mean absolute error 0.19–0.44 in $\log_{10}$ units). For flour, the largest error occurred at 5 g/s, which lies in the widest gap between the five classes (1 to 10 g/s).

Within each velocity, the five flow rates were recorded in ascending order over 45–90 min, so that the class is correlated with recording time. To test whether this order alone allows the class to be predicted, an RBF-SVM was trained on the log-RMS levels of the 13 channels in 1 s no-flow blocks and compared with the same model and features on the flow plateaus. Over the records with a usable no-flow interval before the first feeding cycle, the no-flow level showed no significant trend with class (Spearman ρ=0.29, p=0.33 for flour; ρ=0.06, p=0.82 for coffee). Under leave-one-velocity-out, the no-flow blocks gave 32.8% for flour and 20.4% for coffee (chance 20%), against 57.7% and 54.5% for the flow plateaus; with one no-flow interval of every record held out, as in feeding-cycle hold-out, they reached 44.2% and 35.6%, against 78.1% and 87.7%. Part of this residual information is physical: after the feeding cycles, the flour no-flow level increased with class (ρ=0.58, p=0.02), because powder remaining in the line after 5 and 10 g/s raised it from about 0.3 mV to 0.4–2.0 mV; the remainder reflects record-specific offsets, which may contribute to the accuracy within the trained conditions. Excluding arc segment A_8_.c, with its elevated no-flow level (Section 3.3), lowered the feeding-cycle hold-out balanced accuracy of the RBF-SVM and RF by 0.3–4.8 points but raised their leave-one-velocity-out balanced accuracy for coffee by 6.6–7.3 points, so the noise of this channel also appears to carry record-specific information.

For online use, each 1 s block of 13 channels requires feature extraction and one model evaluation. In an unoptimised single-thread Python implementation on a 2.1 GHz Intel Xeon CPU core, feature extraction took about 68 ms per block and inference 0.10 ms (CVA), 0.17 ms (RBF-SVM), 0.33 ms (MLP) and 99 ms (RF); with CVA, the SVM or the MLP, one second of data is thus processed in less than 0.1 s without a GPU, which is sufficient for real-time monitoring at a 1 s update rate and leaves room for embedded implementation. On an NVIDIA T4 GPU (Google Colab), inference took about 0.4–0.5 ms for the feature-based CNNs and about 0.9 ms for the end-to-end CNN.

## 4. Discussion

Three physical effects, consistent with the observations, may cause the overlap between neighbouring classes (Figure 13); each broadens the signal distribution of a class and matters most where the class levels are closest, between 0.5 and 1 g/s and at 30 m/s. First, because the induced signal depends on the particle–electrode distance as well as on the charge, changes in the cross-sectional solids distribution alter it at the same $Q_{m}$. At S1, only about 30 pipe diameters downstream of the feeder inlet (Section 3.1), the particles may not yet be fully accelerated and dispersed and can form a rope near the wall. At 15 m/s, the lowest tested velocity and only about 1.5 times the velocity below which the powders settled (Section 3.1), part of the powder may still move intermittently near the pipe bottom, decoupling the particle velocity from the nominal air velocity. Second, powder deposited on the wall and in the bends during a test series is partly re-entrained, adding a slowly varying component unrelated to the instantaneous flow rate; for flour, this was observed as a rise in the no-flow level after the 5 and 10 g/s cycles (Section 3.6). Third, the feeder discharge fluctuates within each cycle (Section 3.1) and some batches were discharged in less than 8 s (Section 3.3), so $Q_{m}$ is only an approximate label of the instantaneous rate. The generally lower levels at 30 m/s (Table 3) are not explained by the signal bandwidth moving beyond the 1 kHz front-end pole, as the shorter transit time of faster particles might suggest [9]. In an additional check on the 15 and 30 m/s records, the lag at which the autocorrelation of ring A_0_ falls to 0.5 did not shorten (0.42–0.58 ms for flour at 1 and 10 g/s, 0.20–0.28 ms for coffee at 1 g/s). Separating these effects would require independent measurement of the solids distribution, for example by flow visualisation or tomography; because they depend on the installation, the absolute signal levels are specific to this rig.

Relative to recent data-driven and multisensor measurements of the solids mass flow rate (Table 7), the present work extends the electrostatic approach to 0.1–10 g/s, with a lowest rate about 30 times below the lowest rate of the closest previous study [17]. It also uses a single sensor type, adds a second material and holds out feeding cycles, air velocities and materials in validation. That study regressed the mass flow rate from a similar arrangement with the particle velocities as inputs and all velocities in training [17], and the multisensor and capacitance-based systems were evaluated at their calibrated conditions or at unseen velocity–rate combinations [21,22,25,26]. With complete operating conditions held out, an end-to-end network on a single ring electrode reached low errors for oil-well cement, using about 5000 signal windows from 10 min records per condition [23]. With about 400 blocks per material, the present accuracy dropped for an unseen velocity and material, in agreement with the transfer errors reported for other pipe orientations, diameters and materials [4,24].

Beyond pneumatic conveying, the same approach may provide a basis for classifying the particle content and velocity of gas flows in which direct measurement is difficult, such as the gas path of gas turbines and aero-engines. There, electrostatic sensors already detect debris and ingested particles [38,39], but gas velocities up to about an order of magnitude above the 15–30 m/s tested here, high temperatures and heterogeneous particles would require the electrodes, the front end and the classifiers to be re-qualified.

## 5. Conclusions

The solids mass flow rate was classified into five levels between 0.1 and 10 g/s from an electrostatic ring–arc sensor array with an electrometer-grade front end, for wheat flour and ground coffee at four air velocities. With fixed analysis windows and complete feeding cycles held out of training, the best classifiers reached balanced accuracies of 86.9% (flour) and 89.6% (coffee), with 97.8–100% of predictions within one class. Random block-wise splitting overestimated the accuracies of the best feature-based classifiers by only 0.5–3 points, but a no-flow control showed that record-specific offsets, inseparable from the class under the ascending recording order, may contribute to the accuracy under the trained conditions. The SVMs, MLP and RF performed within a few points of one another under 8-fold cross-validation and feeding-cycle hold-out, and CVA was competitive only for coffee. CNNs trained from scratch on the engineered features offered no advantage at this dataset size; the end-to-end CNN on the raw signals (flour) and the ImageNet-pretrained ResNet-34 (coffee) exceeded the best conventional classifier by only 1–2 points. Arc electrodes were more informative within the trained velocities, ring electrodes were more robust to an unseen velocity, and their combination was best for flour. The same features also gave an approximate continuous estimate, with typical error factors of about 1.3 at the trained conditions and 1.5–2.8 for flow rates withheld from training.

The method is condition-dependent: depending on the classifier, balanced accuracy fell to 47–70% at an unseen air velocity and to 26–59% across materials. A deployed classifier should, therefore, be calibrated for each material and trained over the target velocity range, preferably with the velocity as an input, taken from the drive set-point or measured by cross-correlating upstream and downstream electrodes of the same array [26]. The flow regime was not independently characterised (Section 4), and only one horizontal pipe section was tested; other pipe diameters, higher solids loadings and longer operation, during which wall deposits and humidity alter the charging, should be examined before industrial use. Future experiments should randomise the recording order, add flow rates, particularly between 1 and 10 g/s, to test continuous estimation, and vary temperature and relative humidity, which were kept approximately constant here and which, when used as inputs, reduced the error on a comparable rig [4]. With larger datasets, gated multi-scale temporal networks on the raw signals [23], recurrent and physics-informed models of charge induction, and class-specific regressors selected by the predicted class will be evaluated.

## Figures and Tables

**Table 1:** Definitions of the 29 features extracted from each 1 s block of each channel; the features of the 13 channels form a 377-dimensional vector per block.

No.	Feature	Definition	No.	Feature	Definition
Time domain			Frequency domain		
1.	Root mean sq., RMS	$\sqrt{\frac{1}{N}\sum_{i=1}^{N}x_{i}^{2}}$	17.	Peak amplitude, $A_{p}$	$\max_{k}S_{k}$
2.	Mean, $\bar{x}$	$\frac{1}{N}\sum_{i=1}^{N}x_{i}$	18.	Total power, TTP	$\sum_{k=1}^{K}P_{k}$
3.	Std. deviation, σ	$\sqrt{\frac{1}{N}\sum_{i=1}^{N}{\left(x_{i}-\bar{x}\right)}^{2}}$	19.	Mean frequency, MNF	$\frac{\sum_{k=1}^{K}f_{k}P_{k}}{\sum_{k=1}^{K}P_{k}}$
4.	Skewness, SKW	$\frac{1}{N\sigma^{3}}\sum_{i=1}^{N}{\left(x_{i}-\bar{x}\right)}^{3}$	20.	Spectral entropy, SE	$-\sum_{k=1}^{K}d_{k}\log_{10}d_{k}$
5.	Kurtosis, KRT	$\frac{1}{N\sigma^{4}}\sum_{i=1}^{N}{\left(x_{i}-\bar{x}\right)}^{4}$	21.	Shape factor, SF	$\frac{1}{\bar{S}}\sqrt{\frac{1}{K-1}\sum_{k=1}^{K}{\left(S_{k}-\bar{S}\right)}^{2}}$
6.	Zero crossings, ZC	$\sum_{i=1}^{N-1}\left[\begin{array}{c} \left(x_{i}>0\wedge x_{i+1}<0\right)\\ \vee \;(x_{i}<0\wedge x_{i+1}>0) \end{array}\right]$	Time–frequency domain		
7.	Variance, VAR	$\frac{1}{N}\sum_{i=1}^{N}{\left(x_{i}-\bar{x}\right)}^{2}$	22.	RMS, RMSt_*TF*_	$\sqrt{\frac{1}{MJ}\sum_{m=1}^{M}\sum_{j=1}^{J}C_{m,j}^{2}}$
8.	Peak-to-peak, PP	$\max_{i}x_{i}-\min_{i}x_{i}$	23.	Mean, $\bar{C}$	$\frac{1}{MJ}\sum_{m=1}^{M}\sum_{j=1}^{J}C_{m,j}$
9.	Peak factor, PF	$\frac{\max_{i}\left|x_{i}\right|}{\mathrm{RMS}}$	24.	Variance, VAR_*TF*_	$\frac{1}{MJ-1}\sum_{m=1}^{M}\sum_{j=1}^{J}{\left(C_{m,j}-\bar{C}\right)}^{2}$
10.	Impulse factor, IF	$\frac{\max_{i}\left|x_{i}\right|}{\frac{1}{N}\sum_{i=1}^{N}\left|x_{i}\right|}$	25.	Quantile-freq. ratio, QFR1	$\frac{f_{25}}{f_{75}}$
11.	Clearance factor, CF	$\frac{\max_{i}\left|x_{i}\right|}{{\left(\frac{1}{N}\sum_{i=1}^{N}\sqrt{|x_{i}|}\right)}^{2}}$	26.	Quantile-freq. ratio, QFR2	$\frac{f_{25}}{f_{90}}$
12.	Entropy, $H_{T}$	$-\sum_{k=1}^{50}p_{k}\ln p_{k}$	27.	Quantile-freq. ratio, QFR3	$\frac{f_{50}}{f_{75}}$
13.	Simple sq. int., SSI	$\sum_{i=1}^{N}x_{i}^{2}$	28.	Quantile-freq. ratio, QFR4	$\frac{f_{50}}{f_{90}}$
14.	Waveform length, WL	$\sum_{i=2}^{N}\left|x_{i}-x_{i-1}\right|$	29.	Mean-crossing irreg., MCI	$\frac{\sqrt{{\mathrm{VAR}}_{TF}}}{\bar{C}}$
15.	Willison amp., WAMP	$\sum_{i=2}^{N}\left[\;|x_{i}-x_{i-1}|>0.1\sigma \;\right]$			
16.	Slope sign chg., SSC	$\sum_{i=2}^{N-1}\left[\begin{array}{c} \left(x_{i}>x_{i-1}\wedge x_{i}>x_{i+1}\right)\\ \vee \;(x_{i}<x_{i-1}\wedge x_{i}<x_{i+1}) \end{array}\right]$			

$x_{i}$ (i=1,…,N; N=20,000): samples of the block after mean removal and clipping at ±3 standard deviations of the unclipped block; [·]: 1 if the condition holds and 0 otherwise; $p_{k}$: fraction of samples in the *k*-th of 50 amplitude bins; $S_{k}=\left|X_{k}\right|/N$, $P_{k}=S_{k}^{2}$ and $d_{k}=P_{k}/\sum_{l}P_{l}$: one-sided amplitude spectrum, power spectrum and normalised power spectrum of the *N*-point fast Fourier transform (FFT) $X_{k}$ at frequency $f_{k}$, k=1,…,K; $\bar{S}$: mean of $S_{k}$; $C_{m,j}$: short-time Fourier transform (STFT) magnitude in frame m=1,…,M and frequency bin j=1,…,J; $f_{p}$: frequency below which *p*% of the time-averaged STFT power ($C^{2}$ averaged over frames, DC bin excluded) lies.

**Table 2:** Test matrix. Each test condition $TC_{ij}$ combines air velocity $V_{s}$ (row) and nominal mass flow rate $Q_{m}$ (column) and was recorded for flour and coffee; its record contains three 10 s feeding cycles, whose fixed 7 s windows give up to 21 one-second blocks. * *TC*_21_: flour record excluded (no measurable flow).

	$Q_{m}$ (g/s)				
$V_{s}$ (m/s)	0.1 ($C_{1}$)	0.5 ($C_{2}$)	1 ($C_{3}$)	5 ($C_{4}$)	10 ($C_{5}$)
15	$TC_{11}$	$TC_{12}$	$TC_{13}$	$TC_{14}$	$TC_{15}$
20	$TC_{21}$*	$TC_{22}$	$TC_{23}$	$TC_{24}$	$TC_{25}$
25	$TC_{31}$	$TC_{32}$	$TC_{33}$	$TC_{34}$	$TC_{35}$
30	$TC_{41}$	$TC_{42}$	$TC_{43}$	$TC_{44}$	$TC_{45}$

**Table 3:** Median RMS output (mV) of the 13 conditioned channels in the fixed 7 s analysis windows; 1 mV corresponds to about 83 pA of input current. No-flow level: 0.22–0.54 mV; –: record excluded.

		$Q_{m}$ (g/s)				
Material	$V_{s}$ (m/s)	0.1	0.5	1	5	10
Flour	15	2.32	2.65	4.06	5.82	7.99
	20	–	4.02	5.81	5.91	8.83
	25	2.25	3.22	4.48	5.03	8.93
	30	1.11	2.87	2.59	3.16	6.76
Coffee	15	1.45	1.53	2.36	4.47	9.40
	20	2.15	3.27	3.52	4.96	7.60
	25	2.11	3.09	3.20	5.20	7.33
	30	1.67	2.52	3.06	2.56	3.10

**Table 4:** Balanced accuracy (%) under the three validation protocols (13 conditioned channels, 1 s blocks; chance level 20%): 8-fold, random stratified 8-fold cross-validation; FC, feeding-cycle hold-out; LOVO, leave-one-velocity-out. CVA and SVMs are deterministic; MLP, RF and feature-based CNNs: mean ± standard deviation over five seeds; 224×224 image networks and end-to-end CNN: over three seeds, FC and LOVO only.

	Flour			Coffee		
Model	8-Fold	FC	LOVO	8-Fold	FC	LOVO
CVA	78.4	80.1	51.8	85.5	85.3	55.0
SVM (linear)	82.9	82.5	70.3	80.3	81.8	47.2
SVM (RBF)	85.9	85.4	65.0	88.7	85.5	49.1
MLP	85.8 ± 0.2	84.2 ± 0.3	68.4 ± 1.4	90.6 ± 0.8	88.1 ± 1.2	47.8 ± 0.6
RF	84.3 ± 0.7	84.1 ± 0.3	62.9 ± 0.7	86.2 ± 0.3	83.1 ± 0.3	59.4 ± 0.6
Convolutional networks						
1D CNN (features)	81.4 ± 1.1	79.6 ± 1.7	59.7 ± 2.2	86.4 ± 1.2	84.9 ± 0.6	50.3 ± 1.6
2D CNN (features)	82.4 ± 0.9	76.5 ± 3.0	58.5 ± 3.0	82.3 ± 3.2	77.0 ± 5.9	52.3 ± 2.1
Custom CNN (224×224)	–	80.7 ± 0.2	61.4 ± 0.8	–	84.6 ± 2.3	57.3 ± 0.9
ResNet-34 (224×224)	–	82.9 ± 1.0	62.5 ± 3.1	–	89.6 ± 1.0	57.3 ± 3.1
End-to-end CNN (raw)	–	86.9 ± 0.7	65.8 ± 0.3	–	82.2 ± 1.8	51.2 ± 0.5

**Table 5:** Class-wise precision, recall and F1 score (%) of the RBF-SVM under feeding-cycle hold-out.

		$Q_{m}$ (g/s)				
Material	Metric	0.1	0.5	1	5	10
Flour	Precision	84.1	74.1	75.9	93.9	98.8
	Recall	86.9	78.8	73.2	91.7	96.4
	F1 score	85.5	76.4	74.5	92.8	97.6
Coffee	Precision	89.2	76.2	76.1	90.5	96.3
	Recall	86.8	73.5	80.7	92.7	94.0
	F1 score	88.0	74.8	78.4	91.6	95.1

**Table 6:** Balanced accuracy (%) of the RBF-SVM for ring, arc and combined electrode subsets.

	Feeding-Cycle Hold-Out		Leave-One-Velocity-Out	
Electrodes	Flour	Coffee	Flour	Coffee
Rings (6)	78.9	75.0	63.3	61.7
Arcs (7)	82.8	87.5	57.9	44.6
Combined (13)	85.4	85.5	65.0	49.1

**Table 7:** Recent solids mass flow rate ($Q_{m}$) measurements in gas–solid flows compared with this work. *v*: air or particle velocity; DP: differential pressure; PSD: power spectral density; ECC: electrostatic-coupled capacitance; LSTM: long short-term memory; MAPE: mean absolute percentage error.

Ref.	Sensing	Material; Pipe	$Q_{m}$ (g/s); *v* (m/s)	Model; Evaluation	Reported Result
[17]	4 ring and 16 arc electrodes, DP	Flour (0.21 mm); 50 mm, horizontal, vertical	3.2–35.8; 10.1–36.0	ANN, SVM, CNN regression with velocity input; 70/30 split, six unseen rates (two outside the range), all velocities trained	Max. relative error ±8% (CNN)
[21]	Acoustic, capacitive, electrostatic	Silica sand (77–321 µm); 54 mm square, horizontal, vertical	2.2–7.2; 11–23	CNN–LSTM regression; 9 of 49 velocity–rate combinations unseen (4 outside the range)	Relative error within ±1%
[22]	Electrostatic PSD, cross-correlation velocity	Silica sand (150–200 µm); 50 mm	2.8–6.9; five air settings	PSD–velocity mapping; 20 calibrated conditions	Error below 4%
[26]	ECC, cross-correlation velocity	Polypropylene pellets (3.85 mm); 50, 100 mm	21.8–72.9 (50 mm); 6–7	Velocity × concentration; calibrated at one blower setting, tested at two others	Max. relative error 5.5%
[25]	ECC, spatial-filtering velocity	Sand (≈1 mm); 38 mm inclined, gravity-fed	80–283; gravity flow	Velocity × concentration; four flow rates	Relative error within 10%
[24]	Acoustic emission	Polypropylene, polyethylene pellets; 25, 40 mm	2.0–25 (from mass flux); 3–11	ANN regression; other pipe diameter and material, input standardisation	8.4% within set-up; 16–39% across pipes, 21–43% across materials (>100% unstandardised)
[23]	Single ring electrode	Oil-well cement; DN100 field rig	Industrial (t/h); –	End-to-end temporal network; one of eight tank pressures (0.08–0.22 MPa) held out per fold	MAPE 2.0–4.6%; coefficient of determination 0.97
This work	6 ring electrodes, 7 arc segments, electrostatic only	Flour (200 µm), coffee (300 µm); 50 mm, horizontal	0.1–10; 15–30	CVA, SVM, MLP, RF, CNN classification, SVR and RFR; feeding-cycle, velocity and material hold-out	Balanced accuracy 86.9% and 89.6%, 97.8–100% within one class; 47–70% for an unseen velocity; RFR factor 1.3, 79.2% and 82.1% rounded

## Data Availability

The data presented in this study are available from the corresponding author upon reasonable request.

## Abbreviations

The following abbreviations are used in this manuscript:

AFE	analogue front end
ANN	artificial neural network
BNC	Bayonet Neill–Concelman (connector)
CNN	convolutional neural network
CVA	common vector approach
DAS	data acquisition system
FC	feeding-cycle hold-out
FFT	fast Fourier transform
FNA	flow noise antenna
LOVO	leave-one-velocity-out
MLP	multilayer perceptron
MNF	mean frequency
RBF	radial basis function
ReLU	rectified linear unit
RF	random forest
RFR	random forest regressor
RH	relative humidity
RMS	root mean square
SCC	signal conditioning circuit
STFT	short-time Fourier transform
SVM	support vector machine
SVR	support vector regressor
TC	test condition
TIA	transimpedance amplifier
TRB	triaxial bayonet (connector)
VFD	variable-frequency drive

## Appendix A. Supporting Figures

### Appendix A.1. Signal Conditioning Chain

Figure A1 and Figure A2 give the full frequency and pulse responses of the signal conditioning chain behind the pole frequencies and the pulse distortion quoted in Section 3.2.

### Appendix A.2. Signal Representations

Figure A3 resolves the individual particle pulses behind the spectra of Figure 10, which the STFT of the surrounding 1 s block (Figure A4) averages at the resolution of the time–frequency features (Section 3.3). Figure A5 shows the coherence between neighbouring electrodes, absent from the per-channel features, against its no-flow reference (Section 3.5). At 50 Hz, the mains component gives a ring–arc coherence of 0.8–0.9 at 0.1 g/s and at most 0.16 at 10 g/s; without flow, the coherence is below 0.1 at all frequencies except at 50 Hz (0.6–1.0) and its harmonics. Above the 1 kHz front-end pole, the coherence is below 0.05 in most records, but a broadband coherence of about 0.2–0.35 occurs between the two rings in 9 of the 19 flour records and in one coffee record, and in the flour record at 0.5 g/s also between ring and arc (Figure A5a,c). The common component was absent without flow and persisted after clipping, which argues against a fixed acquisition artefact; its origin remains unresolved, as flow-induced mechanical excitation of the sensor and flow-dependent measurement effects cannot be distinguished with these data.

### Appendix A.3. Feature Relevance and Classifier Diagnostics

Figure A6 gives the per-class distributions of four features of Table 1 for ring electrode A_0_, behind the class trends and the overlap of neighbouring classes reported in Section 3.3.

Under feeding-cycle hold-out, both 224×224 image networks reach their held-out accuracy within the first ten epochs and keep it to the end of training (Figure A7, complementing Figure 12; settings of Section 2.3; the held-out cycle was only monitored, without early stopping or model selection). In a separate single-seed run, the custom CNN of the preliminary analysis reached 80.7% for flour and 86.6% for coffee at epoch 100, and the fully fine-tuned ResNet-34 reached 84.3% and 88.6% at epoch 30 (Table 4 gives the means over the seeds). The held-out loss of the custom CNN rises slowly after about epoch 20 while its accuracy stays level, the usual sign of growing over-confidence on the training cycles rather than of lost discrimination. The ResNet-34 converges faster, its held-out loss staying between 0.4 and 0.9 from the third epoch on.

Figure A8 resolves by feature and channel the Fisher scores that Section 3.5 summarises by electrode type and feature group.
